# CONsensus-based Process evaluation reporting guideline for public HEalth intervention Studies (CONPHES) conducted alongside an effectiveness trial: an e-Delphi study

**DOI:** 10.1136/bmjopen-2024-093962

**Published:** 2025-12-19

**Authors:** Femke van Nassau, Bart Cillekens, Judith G M Jelsma, Christiaan Vis, Lidwine B Mokkink, Shaun Treweek, Hidde P van der Ploeg, e-Delphi panel members, J R Anema

**Affiliations:** 1 Amsterdam UMC, Department of Public and Occupational Health, Amsterdam Public Health research institute, Amsterdam, Netherlands; 2 Physical Activity for Health Research Centre, Moray House School of Education and Sport, University of Edinburgh, Edinburgh, Scotland, UK; 3 Windesheim University of Applied Sciences, Research group Living Well with Dementia, Zwolle, Netherlands; 4 Children's Nutrition Research Center, Baylor College of Medicine, Houston, Texas, USA; 5 Amsterdam University of Applied Sciences, Faculty of Social Work and Law, Amsterdam, Netherlands; 6 University of Amsterdam, Faculty of Social and Behavioural Sciences, Amsterdam, Netherlands; 7 Norwich Medical School, University of East Anglia, Norwich Research Park, Norwich, UK; 8 Institute for Health and Social Care, University of Bradford, Bradford, UK; 9 Wolfson Centre for Applied Health Research, Bradford Royal Infirmary, Bradford, UK; 10 Division of Cancer Control and Population Sciences, National Cancer Institute, Rockville, Washington DC, USA; 11 Centre for Child and Adolescent Mental Health, Eastern and Southern Norway, Section for Infants and Young Children, Oslo, Norway; 12 Physical Activity & Nutrition Determinants in Asia (PANDA) programme, Saw Swee Hock School of Public Health, National University of Singapore and National University Health System, Singapore; 13 Department of Service Research and Innovation, Centre for Child and Adolescent Mental Health, Eastern and Southern Norway, Oslo, Norway; 14 School of Healthcare and Nursing Sciences, Faculty of Health and Wellbeing, Northumbria University, Northumbria, UK; 15 Norwich Medical School, University of East Anglia, Norwich, UK; 16 Netherlands Organisation for Health Research and Development (ZonMw), Department of Innovation and Development, The Hague, Netherlands; 17 School of Nursing, Midwifery and Paramedic Practice, Robert Gordon University, Edinburgh, Scotland, UK; 18 Advanced Care Research Centre, Usher School of Population Health Science, Edinburgh, Scotland, UK; 19 Research Group TRANSFORM’s Idiosynchratic Inventors Collective, Faculty of Social Sciences, KU Leuven, Leuven, Belgium; 20 Director of the Centre for Implementation Trimbos Institute, Utrecht, Netherlands; 21 Institute for Evidence-Based Healthcare, Bond University, Gold Coast, Queensland, Australia; 22 Radboudumc, IQ Health Science Department (IQ Health), Nijmegen, Netherlands; 23 Norfolk County Council, Public Health, Norwich, UK; 24 Physical Activity for Health Research Centre (PAHRC), Institute for Sport, Physical Education and Health Sciences, University of Edinburgh, Edinburgh, UK; 25 Institute for Physical Activity and Nutrition (IPAN), Deakin University, Geelong, Victoria, Australia; 26 Unit of Intervention and Implementation Research for worker health, Institite of Environmental Medicine, Karolinska Institutet, Stockholm, Sweden; 27 Faculty of Medicine, Northern Ontario School of Medicine (NOSM) University, Sudbury, Ontario, Canada; 28 University of South Australia; Alliance for Research in Exercise, Nutrition and Activity (ARENA), Allied Health and Human Performance, South Australia, Adelaide, Australia; 29 Department of Health Research Methodology, McMaster University, Hamilton, Ontario, Canada; 30 Centre for Implementation Research, Clinical Epidemiology Program, Ottawa Hospital Research Institute and School of Epidemiology and Public Health, University of Ottawa, Ottowa, Ontario, Canada; 31 School of Public Health, University College Cork, Cork, Ireland; 32 Institute for Health Services Research and Health Economics, Centre for Health and Society, Medical Faculty and University Hospital Düsseldorf, Heinrich-Heine-University Düsseldorf, Düsseldorf, Germany; 33 Academy of Health and Welfare, Halmstad University, Halmstad, Sweden; 34 Health and Care Research Unit, Sheffield Centre for Health and Related Research (SCHARR), University of Sheffield, Sheffield, UK; 35 Department of Nutrition, Institute of Basic Medical Sciences, University of Oslo, Oslo, Norway; 36 University of Leeds, Leeds, UK; 37 Usher Institute, The University of Edinburgh, Edinburgh, UK; 38 Northumbria University, School of Communities and Education, Northumbria, UK; 39 Center for Mental Health Services Research, Brown School, Washington University in St. Louis, St Louis, Missouri, USA; 40 School of Public Health, Washington University in St. Louis, St. Louis, Missouri, USA; 41 School of Communities and Education, Faculty of Health and Wellbeing, Northumbria University, Northumbria, UK; 42 Department of Kinesiology & Nutrition Sciences, University of Nevada, Las Vegas, Nevada, USA; 43 Mälardalen University, School of Health, Care and Social Welfare, Västerås, Sweden; 44 University of Arkansas for Medical Scinces, Department of Pediatrics, Little Rock, AR, USA; 45 Vice President Research, St Joseph's Healthcare--Hamilton Scientific Director, Research Institute of St Joe's Hamilton Professor, Department of Health Research Methods, Evidence, and Impact McMaster University, Hamilton, Ontario, Canada; 46 The University of Sydney, Sydney, New South Wales, Australia; 47 School of Primary Care, Population Sciences and Medical Education, University of Southampton, Southampton, UK; 48 Amsterdam UMC, Vrije Universiteit Amsterdam, Department of Ethics, Law and Humanities, Amsterdam, Netherlands; 49 University of Groningen, University Medical Center Groningen, UMC Staff Policy and Management support, Groningen, Netherlands; 50 University of South-Eastern Norway, Borre, Norway; 51 Amsterdam Collaboration on Health & Safety in Sports, Department of Public and Occupational Health, Amsterdam Movement Sciences, Amsterdam UMC, University Medical Centres – Vrije Universiteit Amsterdam, Amsterdam, Netherlands; 52 Department of General Practice and Health Services Research, Heidelberg University, Heidelberg, Germany; 53 School of Medicine and Public Health, The University of Newcastle, Newcastle, Newcastle, Australia; 54 Swiss Tropical and Public Health Insittute, University of Basel, Allschwil, Switzerland; 55 Centre for Healthcare Innovation Research (CHIR), University of London, London, Northampton Square, UK; 1 Department of Public and Occupational Health and Amsterdam Public Health Research Institute, Amsterdam UMC Locatie VUmc, Amsterdam, Noord-Holland, the Netherlands; 2 Haukeland, Section for Research-Based Innovation, Division of Psychiatry, Haukeland University Hospital, Bergen, Norway; 3 Department of Epidemiology and Data Science and Amsterdam Public Health Research Institute, VU University Medical Centre Amsterdam, Amsterdam, the Netherlands; 4 Health Services Research Unit, University of Aberdeen, Aberdeen, UK

**Keywords:** Delphi Technique, Implementation Science, PUBLIC HEALTH

## Abstract

**Objectives:**

Many researchers conduct a process evaluation alongside an effectiveness trial of a public health intervention to better understand mechanisms behind observed effects. Yet, there is no standardised, scientifically accepted guideline for reporting such process evaluations, which impedes interpretation and comparison of study results. The aim of this project was to develop a consensus-based and expert-based guideline for reporting process evaluations of public health interventions conducted alongside an effectiveness trial.

**Design and setting:**

We conducted an e-Delphi study with a large panel of international experts.

**Participants:**

Based on purposive sampling, we invited 137 international experts that had been involved in the design of process evaluations, researchers who published high-profile process evaluations or frameworks, editors of journals that publish process evaluations, and authors of other reporting guidelines.

**Results:**

Based on a literature search, a first draft of the reporting guideline included 32 items, which was proposed to panel members during the first round. Of the invited 137 invited international experts, 73 (53%) participated in at least one round of the e-Delphi study. Participants rated the inclusion and comprehensibility of the proposed items on a 5-point Likert scale and provided comments and suggestions for relevance and definitions of the items. Adjustments to the items and descriptions were proposed to the e-Delphi panel until consensus of ≥67% for each individual item was reached. In total, 64 (88% of 73) completed round 2, and 55 (76% of 73) completed round 3. This resulted in 19 items that are included in the consensus-based process evaluation reporting guideline for public health intervention studies (CONPHES) guideline. The items cover a detailed description of the intervention that is evaluated, the implementation strategies applied, and underlying causal pathways, and the role of the delivery and support team. The guideline also requires describing the evaluation framework and how evaluation outcomes were assessed. Lastly, the guideline includes items on providing a detailed description of applied analyses (both quantitative and qualitative) and measures for assuring quality. The guideline is accompanied by an Explanation and Elaboration document, with a more detailed explanation of each item.

**Conclusions:**

We expect that the CONPHES reporting guideline for process evaluations of public health interventions can improve the reporting of process evaluations of interventions aimed at promoting public health. This can potentially facilitate more effective translation of public health research into practice and contribute to improving both individual and population health outcomes.

**STRENGTHS AND LIMITATIONS OF THIS STUDY**

- A strength of this study was the structured e-Delphi approach to gather input and feedback on items for inclusion for the guideline.
- We engaged a large international panel, though limited participation from low-income and middle-income countries may have biased results.
- Despite the rigorous consensus process, some relevant items or nuances in definitions and descriptions may have been missed.

## Introduction

 Conducting a process evaluation has become more popular; the number of published articles has roughly quadrupled in PubMed from 162 in 2012 to 661 in 2022. Process evaluations of public health interventions are essential for understanding how impact is (not) achieved in practice and to understand the interplay between implementation, intervention mechanisms and context in which these impacts are achieved.^1^ With a process evaluation, researchers may aim to examine the perspectives of participants regarding the intervention; for example, study how the intervention is implemented, distinguish between components of the intervention, investigate contextual factors that affect implementation of an intervention, and monitor dose and assess the reach of the intervention. Such an investigation offers insights into how these factors might have impacted the outcomes of an effectiveness trial,^1 2^ looking at the intervention effect on the target population. Therefore, researchers can conduct a process evaluation alongside an effectiveness trial of public health intervention studies.

Although there are many frameworks, theories and models for designing process evaluations, such as the RE-AIM framework,^3^ UK-MRC process evaluation model^1 4^ and Durlak and Dupre’s model,^5^ reporting of process evaluations is often inadequate and incomplete and there is a wide variety in how process evaluations are conducted.^6^ In order to enhance reporting processes, the EQUATOR (Enhancing the QUAlity and Transparency Of health Research) Network was initiated to stimulate the development of such guidelines, and also provide an overview of existing guidelines.^7^ Reporting guidelines aim to improve transparency, replication and comparisons across studies.^8^ In the field of health intervention studies, many reporting guidelines have been developed and are being used in practice, such as the consolidated standards of reporting trials (CONSORT) guideline^9^ and its extensions for pragmatic trial reporting,^10^ TIDieR Checklist with regard to intervention descriptions,^11^ Strengthening the reporting of observational studies in epidemiology guideline for observational studies,^12^ COREQ or SRQR guidelines for reporting qualitative research,^13 14^ and the Standards for Reporting Implementation Studies (StaRI).^15^

However, there is currently no standardised, scientifically accepted guideline for reporting process evaluations conducted alongside an effectiveness trial. This hinders transparency in the interpretation of results and comparison between trials. Therefore, the aim of this project was to develop a consensus-based and expert-based guideline for reporting process evaluations of public health intervention studies conducted alongside an effectiveness trial.

## Methods

### Study design

We conducted an e-Delphi study with three rounds of expert input between October 2020 and January 2022. The e-Delphi method is a series of three sequential ‘rounds’, interspersed by controlled feedback that seeks to achieve consensus of opinion among the panel participants.^16^ We considered the e-Delphi technique as the most suitable consensus method, as participants could participate online, increasing the likelihood of participation. Our full protocol is available on the EQUATOR website.^17^ We did make some changes to the published protocol: we applied a 67% (vs 80%) threshold for consensus in line with other studies^18^ and used a 5-point Likert scale instead of a 9-point scale. We were also able to recruit more panel members than anticipated (73 vs the 30 we had planned). We used the standard for Conducting and Reporting of Delphi Studies^19^ to design and report the study (see online supplemental appendix 1).

### Steering group

We created a steering group to guide the development process. This group consisted of the authors (FN, BC, JGMJ, CV, LBM, ST and HPP), with experience in conducting process evaluations, setting up trials, developing reporting guidelines, conducting e-Delphi studies and in the role of editor. This group conducted a literature search to identify existing checklists used to assess or review process evaluation studies, potential reporting guidelines for process evaluations and systematic reviews of process evaluation studies. The steering group also developed the rounds of the e-Delphi study. Feedback after each round was discussed with the group and proposals for an alternative were formulated to inform the next round. The steering group members had no vote in the e-Delphi rounds.

### Recruitment of panel members

We applied a purposive sampling procedure for inviting international experts.^20^ In the first step, we reached out to researchers that had been involved in the design of process evaluations, researchers who published high-profile process evaluations or frameworks, editors of journals that publish process evaluations, and authors of other reporting guidelines. The results of the literature search and existing EQUATOR network guidelines helped to identify potential panel members. In the second step, the steering group members could provide further suggestions for eligible panel members. Finally, panel members were encouraged to propose peers with expertise in process evaluations of public health intervention studies conducted alongside an effectiveness trial.

### Preparation

In May 2020, we conducted a search for existing reporting guidelines for process evaluations or similar research designs that mostly concern qualitative or mixed methods research. The aim of the search was to identify potential items and descriptions from other reporting guidelines to inform the first draft of items for Round 1. We performed a literature search using PubMed (online supplemental appendix 2), to identify checklists used to assess or review process evaluation studies, and potential existing guidelines for reporting process evaluation studies, systematic reviews of process evaluation studies, and reference lists of relevant publications. In addition, we included relevant reporting guidelines that were published at the EQUATOR network website.^7^

In total, 14 guidelines were found.^911^,^15 21^ From these guidelines, we extracted potentially relevant items to inform the creation of our new process evaluation guidelines. We (FN, BC, JGMJ, CV) then assessed these existing guidelines to identify potential domains and items for the new checklist. We discussed among the steering group which items we would propose to be included in the consensus-based process evaluation reporting guideline for public health intervention studies (CONPHES) reporting guideline.

### e-Delphi procedure

Eligible participants were invited by a personalised email to participate in the e-Delphi study. We informed them that participation was voluntary and would take approximately 30–60 min each round. Per round, two email reminders were sent bi-weekly. We used Survalyzer software (http://www.survalyzer.com/nl/) to facilitate the web-based process and pretested each round among researchers not partaking in the e-Delphi study.

In each round, we asked panel members to rate the inclusion and comprehensibility of the proposed items on a 5-point Likert scale. Responses were analysed anonymously. Consensus was considered to be reached on items when at least 67% of the panel members scored ‘agree’ or ‘strongly agree’ on the five-point Likert scale.^18^ If <67% agreement was reached for an item or description, it was revised based on the feedback and included in the next e-Delphi round.

In addition, we asked panel members to provide their comments and suggestions for improving the descriptions of the items. These suggestions allowed us to improve the items and make more specific proposals in the next round. Free-text responses were initially analysed independently by FN and BC, who extracted and grouped the main points. Remaining discussion points were then presented to the steering group for deliberation and agreement on any changes to the items. In the subsequent e-Delphi round, participants were informed of the changes and provided with explanations regarding how their feedback had been incorporated. Items for which a consensus on inclusion was not achieved in the previous round were carried forward to the subsequent round. These items were accompanied by the presented pros and cons, along with an alternative formulation/phrasing.

When consensus on a particular item was not reached after three rounds, the steering group made the final decision based on arguments provided to either exclude, adapt or integrate the item in other items.

### Exploration and elaboration (E&E) document

Based on the input provided by the participants in the three rounds of the e-Delphi study, an Exploration and Elaboration (E&E) document was developed to accompany the checklist. This document was drafted by FN, BC and CV, and reviewed by the steering group. Lastly, a draft of the E&E document was reviewed by 12 potential guideline users (ie, mix of early career and mid-career researchers working in the public health intervention field), during a workshop held in April 2023. Feedback during this workshop was used to improve usability of the guideline and to improve explanatory texts.

## Results

Of the 137 experts invited, 72 participated in round 1, 64 in round 2 (including one participant who did not take part in round 1), and 55 completed round 3 (67%)—who are listed as **e-Delphi panel members**. Seven experts indicated that they did not want to participate. For two experts, the email address seemed to be inactive. The remaining experts (n=56) did not respond.

A description of the panel members can be found in table 1. From the 73 participants, 47% had more than 11 years of experience in conducting and reporting process evaluations. With regard to professional background, 85% of participants were health researchers, 23% methodologists, 10% journal editors and 4% guideline developers. Participants could select more than one professional background. Of the 73 participants who took part in at least one e-Delphi round, the vast majority were based in Europe (65%) and North America (23%), with a smaller number from Oceania (8%). Participation from Africa and Asia was limited (4%). This geographical distribution reflects the composition of the expert panel and is noted as a limitation in the discussion. A flowchart of e-Delphi panel members participating in each round can be found in online supplemental appendix 3.

**Table 1:** Description of the panel members of the e-Delphi (n=73)

		n=73 (%)
Gender	Female	49 (67)
	Male	24 (33)
Continent usually working from*	Europe^†^	49 (65)
	North America^‡^	17 (23)
	Oceania^§^	6 (8)
	Africa^¶^	2 (3)
	Asia^**^	1 (1)
	Not specified	1 (1)
	Currently unemployed	1 (1)
Professional background/expertise^††^	(Public) health researcher	62 (85)
	Methodologist	17 (23)
	Journal Editor	7 (10)
	Healthcare professional	5 (7)
	Guideline developer	3 (4)
	Manager	2 (3)
	Implementation expert	2 (3)
Years of experience with reporting process evaluations	0–5 years	14 (19)
	6–10	25 (34)
	11–15	11 (15)
	16+	23 (32)

* Some participants indicated multiple countries, n=77 in total.

† UK (N=21), the Netherlands (N=10), Sweden (N=3), Norway (N=5), Ireland (N=3). Germany (N=2), France, Denmark, Switzerland, Portugal, Belgium (N=1).

‡ US (N=9), Canada (N=8).

§ Australia (N=5), New Zealand (N=1).

¶ Ghana, Tanzania (N=1).

** Singapore (N=1).

†† Partipcants could indicate more than one expertise.

### e-Delphi study

#### Round 1

In Round 1 of the e-Delphi study (October 2020–November 2020), we proposed 32 items and their descriptions to all panel members (online supplemental appendix 4). The items were divided into three domains: (1) Intervention and implementation context, (2) Study design and (3) Analysis and findings. In round 1, 72 respondents participated. One respondent could not participate in round 1, but this participant was willing to participate in the other rounds and was therefore included in round 2. Consensus was reached for the inclusion of 26 (81%) items and for the ease of understanding of the item description of 11 items (34%) in this first round (see online supplemental appendix 4).

With regard to feedback on the structure of the guideline, many panel members suggested sticking to the CONSORT domains (ie, introduction-methods-results-discussion).^9^ Multiple panel members expressed that the item list was too long and too detailed, potentially reducing the usability of the guideline. Furthermore, it was questioned whether certain items were specific for a process evaluation or more generally applicable to any evaluation study. Based on this feedback, we reordered the items into four main domains that correspond to the common structure of a scientific manuscript (ie, introduction-methods-results-discussion). We critically reviewed all items conceptually and simplified and merged items where possible. Only items specific to reporting process evaluations were retained.

#### Round 2

The changes resulted in a reduction from 32 to 19 items and reduced the level of detail required for each item in Round 2. The adapted list of 19 items was circulated to panel members during the second round of the e-Delphi study (April 2021–June 2021). The 4 items and two descriptions for which consensus was reached were included for reference and participants were not asked to rate them again (see online supplemental appendix 5).

We received complete responses in Round 2 from 64 (88%) of the 73 panel members. Consensus was reached on 17 of 19 items and 16 of the 19 descriptions (see online supplemental appendix 5). In general, panel members were satisfied with the changes. The scope was clearer, the format improved and the number of items was more manageable.

#### Round 3

In round 3 (December 2021–January 2022; see online supplemental appendix 6), we asked panel members to only rate the remaining and amended two items, and three descriptions, as consensus was already reached on all other items and descriptions. The panel members were able to review the complete guideline and provide general comments and suggestions on the comprehensiveness of the items and their descriptions on which consensus had already been reached. In total, 55 of 64 participants who took part in round 2 (86%) completed round 3. Consensus was reached on all item definitions and descriptions, except for the description of one item (unexpected factors; see online supplemental appendix 6, where 60% of panel members agreed. The steering group discussed the remaining feedback and suggestions for this item after round three and revised the description accordingly.

### The CONPHES checklist

The final CONPHES reporting guideline consists of 19 items (see table 2). Included items consist of Title/abstract (one item), Introduction (one item), Methods (12 items), Results (two items) and Discussion (three items). Items range from a comprehensive description of the intervention, to the implementation strategies applied, and the role of the delivery and support team. The guideline also includes items for describing the evaluation framework and assessment of evaluation outcomes. Lastly, a number of items relating to the analytical framework and analyses applied (both quantitative and qualitative) and the measures for quality assurance are included in the guideline.

**Table 2:** The consensus-based process evaluation reporting guideline for public health intervention studies guideline

Item number	Item	Description
Title/abstract		
1	Title and abstract	Describe in the title and/or abstract and keywords that the study includes a process evaluation
Introduction		
2	Process evaluation aim(s) and/or objective(s)	Describe aim(s) and/or objective(s) of the process evaluation study
Methods		
Intervention		
3	Description of the intervention	Describe (1) The intervention development process, with reference to, for example, previous research, frameworks and co-creation that were used in the process of developing the intervention, (2) The intervention, such as the aim of the intervention, components/activities, target population, materials for participants, mode of delivery (eg, face-to-face, online, individual, group-based), duration and frequency and (3) If the intervention was pilot tested. *If applicable, refer to other documents that describe the intervention*
4	Implementation strategies	Describe implementation strategies (methods or techniques) used to enhance the adoption, implementation and sustainability of the intervention such as materials to support intervention delivery, training of intervention deliverers, organisational support or intervention delivery resources *If applicable, refer to other documents that describe the implementation strategies*
5	Mechanisms of the intervention	Describe the mechanisms by which the intervention and implementation strategies are expected to achieve the desired effects. *If applicable, present a theory of change or logic model*
6	Delivery and support team	If applicable, describe who delivered the intervention and implementation strategies, and their expertise such as skills, qualifications and prior experience in delivering the intervention or implementation strategies. *If applicable, describe any potential role members of the research team had*
7	Context	Describe the context in which the intervention was implemented, such as the type(s) of location(s) or setting(s) where the intervention was delivered, the physical and geographical environment(s), and the socio-cultural and socio-economic context(s)
Process evaluation design		
8	Process evaluation framework	Describe the theory, model or framework guiding the process evaluation
9	Process evaluation outcome(s)	Describe any outcome(s) that were assessed in the process evaluation and how they were defined and operationalised. *If assessed, describe process evaluation outcomes such as reach, adoption, recruitment, fidelity, adherence, dosage (delivered and received), quality of delivery, programme differentiation, adaptation, satisfaction, responsiveness, maintenance, costs, facilitators and barriers for implementation*
Data collection		
10	Recruitment for the process evaluation	Describe how, by whom, and when process evaluation participants (eg, target population, intervention deliverer, location/setting) were recruited, and provide a rationale for the recruitment strategy. *If applicable, describe any eligibility criteria and/or incentives used for participant enrolment.* *If applicable, describe any eligibility criteria for the location(s) or setting(s*)
11	Process evaluation data collection procedures	Describe (1) What data sources were used (eg, questionnaire, interview, focus group, observation, routine collected data, field notes), (2) Which of the process evaluation outcomes they relate to, (3) the timing of process data collection (eg, at the study start, during and at the end of the process evaluation study) and (4) Data collection setting(s). *If applicable, describe who collected process data, their role in the study and their relationship with study participants (eg, target population, intervention deliverer).* *If applicable, describe whether pre-existing methods and instruments were used or whether they were developed specifically for this study*
12	Control group data collection	Describe if any process evaluation data were collected among participants in the control or comparison group(s)
Analysis		
13	Process evaluation data analysis	Describe (1) Data analysis (ie, qualitative, quantitative and/or mixed methods), (2) Who analysed the data and (3) If the results of the effectiveness study were known at the time of process data analysis. *If applicable, name and describe the software used to facilitate the analysis.* *If applicable, describe how data integration techniques were conducted to integrate quantitative and qualitative data.* *If applicable, describe if the relationship between process evaluation findings and effectiveness outcomes was assessed (eg, dose-response analysis*)
14	Quality assurance of data analysis	Describe techniques that were used to ensure the quality of qualitative and/or quantitative data analysis. *If applicable, for qualitative analyses, describe processes such as member checking, audit trail and triangulation. For quantitative analyses, describe processes such as data cleaning, blinding and how missing data were handled*
Results		
15	Characteristics of process evaluation participants	Describe characteristics of participants in the process evaluation, and, where relevant, the intervention deliverer(s) and setting(s), and describe to what degree the process evaluation participants and setting(s) were representative of the target population of the effectiveness trial. *If assessed, describe characteristics of the control or comparison group participants*
16	Results of process evaluation	Describe the results of the process evaluation as defined in the methods section (as listed in items 9 and 13)
Discussion		
17	Interpretation of findings	Describe how the findings of the process evaluation can be interpreted. *If applicable, describe how effectiveness outcomes should be interpreted in light of the process evaluation findings*
18	Unexpected factors	Describe previously unforeseen factors that may have influenced the implementation of the intervention and/or the process evaluation results
19	Implications	Discuss implications of the process evaluation outcomes for future intervention delivery, and implications for policy, practice and/or research

The full guideline is included in online supplemental appendix 7. Details about each of the items are provided in the Explanation and Elaboration (E&E) document, in which examples are provided (online supplemental appendix 8).

## Discussion

The aim of this study was to develop a consensus-based and expert-based guideline to improve the reporting quality of process evaluations from public health intervention studies conducted alongside an effectiveness trial. Through an e-Delphi study, we developed the final guideline consisting of 19 reporting items. The CONPHES guideline is registered with the EQUATOR Network (www.equator-network.org),^7^ and the complementary Checklist (for completion by authors) can be found in online supplemental appendix 7.

There are several immediate applications of this guideline: it can assist authors who are writing a process evaluation manuscript, or those writing the protocol for their planned process evaluation. Furthermore, the guideline can be used by editors and reviewers to check if all necessary process evaluation information is described. As such, we invite editors of journals publishing process evaluations to make the CONPHES guideline obligatory for new submissions. We do acknowledge that reporting on all items might not be feasible due to word limits of journals. In such cases, for example, detailed intervention mechanisms and implementation strategy descriptions could be included in an appendix. It is strongly recommended that authors using the CONPHES guideline read the detailed E&E document that explains the rationale for each item and provides examples of good practice. Furthermore, the guideline can also be relevant to other types of process evaluations, including retrospective evaluations or evaluations without an effectiveness study.

Based on feedback from the panel members, we have clarified that the guideline focuses on process evaluations conducted in the context of effectiveness trials. We have also chosen to focus on process evaluations of public health interventions because these tend to be complex behavioural interventions, for which process evaluations are useful methods for improving understanding of intervention processes to explain (lack of) effects. And no available reporting guidelines were available. We also did not aim to replace existing guidelines and recognise that some parts of the reporting guideline can be complemented with other existing guidelines, such as TIDieR Checklist with regard to intervention descriptions^11^ or the StaRI.^15^ We, therefore, have referred to such guidelines in the E&E document.

As process evaluations may apply different methodologies, such as using predominantly quantitative or qualitative methods or using mixed methods. We do not provide recommendations or advocate for specific approaches, methods or quality standards. Yet, the guideline can facilitate identifying weaknesses in the reporting of a manuscript by making reports more explicit and transparent.

### Strengths and limitations

A strength of this study was that we were able to gather input from a large panel of experts. Building on the potential panel members identified in the literature search, snowball sampling helped to expand the panel. However, there were few experts from low/middle-income countries participating in the study, which might have skewed the findings towards research settings in high-income countries.

It needs to be mentioned that the panel members were very engaged in the rigorous consensus process, as 67% completed all rounds, and we reached consensus on all 19 items, and also consensus on all but one description of these 19 items. Also, the amount and quality of the feedback we received from the majority of participants was high. Many valuable comments and suggestions were made by the panel members. It may be possible that despite the rigorous consensus process, we have missed potentially relevant items or nuances in definitions and descriptions. Therefore, we welcome suggestions for further improvement.

A final strength of this study was the pre-study registration of the study protocol for the e-Delphi^8^ on the EQUATOR website.^7^ The e-Delphi study and drafting of the manuscript and E&E document took more time than anticipated, so our timeline was delayed compared with what we had planned in the study protocol. Yet, we were able to include more experts in the panel, take more time to review and reflect on the rounds, and incorporate feedback on the E&E document with potential users during a workshop, all contributing to the quality of the guideline.

A limitation of our study is the geographical distribution of the expert panel. Of the 73 participants who took part in at least one e-Delphi round, the vast majority were based in Europe and North America, with a smaller number from Oceania. Participation from Africa and Asia was limited, representing <5% of the total panel. While the panel included diverse professional backgrounds, the underrepresentation of experts from low-income and middle-income countries may affect the generalisability of the guideline to these settings.

### Conclusion

This consensus-based e-Delphi study has resulted in the comprehensive CONPHES reporting guideline for researchers and practitioners involved in conducting and reporting public health process evaluations. We hope that the implementation of this guideline will result in higher quality reporting of process evaluations of interventions aimed at promoting public health. This could improve transparency, replication and translation of research into practice and improving the health of individuals and populations.

## Supplementary material

10.1136/bmjopen-2024-093962 online supplemental file 1

10.1136/bmjopen-2024-093962 online supplemental file 2

## Data Availability

No data are available.
