# Supplementary material for: CONsensus-based Process evaluation reporting guideline for public HEalth intervention Studies (CONPHES) conducted alongside an effectiveness trial: an e-Delphi study
Source: BMJ Open. 2025 Dec 19;15(12):e093962. doi: 10.1136/bmjopen-2024-093962 (PMC12716498; doi:10.1136/bmjopen-2024-093962)
Supplement: online supplemental file 1 [file bmjopen-15-12-s001.docx]

**The following authors each** completed all three rounds of the e-Delphi-study*, and provided valuable input for the development of the CONPHES reporting guideline:

| # | e-Delphi Panel Member | Affiliation |
| --- | --- | --- |
| 1 | Anema, Johannes R. | Amsterdam UMC, Department of Public and Occupational Health, Amsterdam Public Health research institute, Van der Boechorststraat 7, 1081 BT Amsterdam, The Netherlands. |
| 2 | Baker, Graham | Physical Activity for Health Research Centre, Moray House School of Education and Sport, University of Edinburgh, Holyrood Road, Edinburgh, EH8 8AQ, Scotland, United Kingdom. |
| 3 | Bakker, Franka C. | Windesheim University of Applied Sciences, Research group Living Well with Dementia, Campus 2-6, 8000 GB Zwolle , The Netherlands. |
| 4 | Baranowski, Tom | Children's Nutrition Research Center, Baylor College of Medicine, Houston, Texas, USA. |
| 5 | Boendermaker, Leonieke | Amsterdam University of Applied Sciences, Faculty of Social Work and Law, PO.Box 1025, 1000 BA Amsterdam, The Netherlands and University of Amsterdam, Faculty of Social and Behavioural Sciences, Nieuwe Achtergracht 127, 1018 WS Amsterdam, The Netherlands. |
| 6 | Burke, Amanda | Norwich Medical School, University of East Anglia, Norwich Research Park, Norwich NR4 7TJ, United Kingdom. |
| 7 | Chalkley, Anna E. | Institute for Health and Social Care, University of Bradford, Bradford, UK; and Wolfson Centre for Applied Health Research, Bradford Royal Infirmary, Bradford, United Kingdom. |
| 8 | Chambers, David A. | Division of Cancer Control and Population Sciences, National Cancer Institute, Rockville, MD 20850, USA. |
| 9 | Drozd, Filip | Centre for Child and Adolescent Mental Health, Eastern and Southern Norway, Section for Infants and Young Children, PO Box 4623 Nydalen, 0405 Oslo, Norway. |
| 10 | Edney, Sarah M. | Physical Activity & Nutrition Determinants in Asia (PANDA) programme, Saw Swee Hock School of Public Health, National University of Singapore and National University Health System, 12 Science Drive 2, #10-01, 117549, Singapore. |
| 11 | Engell, Thomas | Department of Service Research and Innovation, Centre for Child and Adolescent Mental Health, Eastern and Southern Norway, Gullhaugveien 1-3, 0484 Oslo, Norway. |
| 12 | Finch, Tracy | School of Healthcare and Nursing Sciences, Faculty of Health and Wellbeing, Northumbria University, NE1 8ST, United Kingdom. |
| 13 | Fynn, Judith | Norwich Medical School, University of East Anglia, Norwich, NR4 7TJ, United Kingdom. |
| 14 | Goense, Pauline B. | Netherlands Organisation for Health Research and Development (ZonMw), Department of Innovation and Development, Laan van Nieuw Oost-Indië 334, 2593 CE The Hague, The Netherlands. |
| 15 | Grant, Aileen | School of Nursing, Midwifery and Paramedic Practice, Robert Gordon University, Garthdee Road, Aberdeen, AB10 7QB, United Kingdom. |
| 16 | Guthrie, Bruce | Advanced Care Research Centre, Usher School of Population Health Science, Usher Building, 5 Little France Drive, Edinburgh, EH16 4UX, Scotland. |
| 17 | Hannes, Karin | Research Group TRANSFORM’s Idiosynchratic Inventors Collective, Faculty of Social Sciences, KU Leuven, Parkstraat 45, 3000 Leuven, Belgium. |
| 18 | Hipple Walters, Bethany | Director of the Centre for Implementation Trimbos Institute, Da Costakade 45, 3521 VS Utrecht, The Netherlands. |
| 19 | Hoffmann, Tammy C. | Institute for Evidence-Based Healthcare, Bond University, Gold Coast, Australia. |
| 20 | Hulscher, Marlies E.J.L. | Radboudumc, IQ Health Science Department (IQ Health)  Postbus 9101, 6500 HB Nijmegen (160), The Netherlands. |
| 21 | Jones, Andy P. | Norfolk County Council, Public Health, 3rd Floor County Hall, Martineau Lane, Norwich NR1 2DH, United Kingdom. |
| 22 | Kelly, Paul | Physical Activity for Health Research Centre (PAHRC), Institute for Sport, Physical Education and Health Sciences, University of Edinburgh, United Kingdom. |
| 23 | Koorts, Harriet | Institute for Physical Activity and Nutrition (IPAN), Deakin University, Geelong, Australia. |
| 24 | Kwak, Lydia | Unit of Intervention and Implementation Research for worker health, Institite of Environmental Medicine, Karolinska Institutet, Sweden. |
| 25 | Maar, Marion | Faculty of Medicine, Northern Ontario School of Medicine (NOSM) University, 935 Ramsey Lake Road, Sudbury, Ontario, P3E 2C6, Canada. |
| 26 | Maher, Carol A. | University of South Australia; Alliance for Research in Exercise, Nutrition and Activity (ARENA), Allied Health and Human Performance; Australia. |
| 27 | Mbuagbaw, Lawrence | Department of Health Research Methodology, McMaster University, 1280 Main Street West, Hamilton, Ontario, L8S 4L8, Canada. |
| 28 | McCleary, Nicola | Centre for Implementation Research, Clinical Epidemiology Program, Ottawa Hospital Research Institute, Ottawa, ON, Canada; and School of Epidemiology and Public Health, University of Ottawa, Ottawa, ON, Canada. |
| 29 | McHugh, Sheena | School of Public Health, University College Cork, Western Rd, Cork, Ireland. |
| 30 | Milton, Karen | Norwich Medical School, University of East Anglia, United Kingdom. |
| 31 | Möhler, Ralph | Institute for Health Services Research and Health Economics, Centre for Health and Society, Medical Faculty and University Hospital Düsseldorf, Heinrich-Heine-University Düsseldorf, Moorenstraße 5, 40225 Düsseldorf, Germany. |
| 32 | Neher, Margit | Academy of Health and Welfare, Halmstad University, Kristian IV:s väg 3, 301 18 Halmstad, Sweden. |
| 33 | O Cathain, Alicia | Health and Care Research Unit, Sheffield Centre for Health and Related Research (SCHARR), University of Sheffield, Sheffield, United Kingdom. |
| 34 | Paulsen, Mari Mohn | Department of Nutrition, Institute of Basic Medical Sciences, University of Oslo, Box 1110 Blindern, 0317 Oslo, Norway. |
| 35 | Pawson, Ray | University of Leeds. United Kingdom. |
| 36 | Pinnock, Hilary | Usher Institute, The University of Edinburgh, Usher Building, 5-7 Little France Road, Edinburgh BioQuarter, EH16 4UX, Edinburgh. |
| 37 | Potthoff, Sebastian | Northumbria University, School of Communities and Education, Coach Lane Campus West, NE7 7XA, Newcastle upon Tyne, United Kingdom. |
| 38 | Powell, Byron J. | Center for Mental Health Services Research, Brown School, Washington University in St. Louis, One Brookings Drive, St. Louis, Missouri 63105, USA; School of Public Health, Washington University in St. Louis; Division of Infectious Diseases, John T. Milliken Department of Medicine, Washington University School of Medicine; School of Public Health, College of Medicine & Health, University College Cork; School of Population Health, UNSW Medicine & Health, University of New South Wales. |
| 39 | Rapley, Tim | School of Communities and Education, Faculty of Health and Wellbeing, Northumbria University, NE1 8ST, United Kingdom. |
| 40 | Rosenkranz, Richard R. | Department of Kinesiology & Nutrition Sciences, University of Nevada, Las Vegas, 4505 S Maryland Pkwy, Las Vegas, NV 89154, USA. |
| 41 | von Thiele Schwarz, Ulrica | Mälardalen University, School of Health, Care and Social Welfare, Box 883, 721 23 Västerås, Sweden. |
| 42 | Massey-Swindle, Taren | Little Rock, ARUniversity of Arkansas for Medical Scinces, Department of Pediatrics, 15 Children’s Way, Little Rock, AR 72205, USA. |
| 43 | Thabane, Lehana | Vice President Research, St Joseph's Healthcare--Hamilton Scientific Director, Research Institute of St Joe's Hamilton Professor, Department of Health Research Methods, Evidence, and Impact McMaster University, Hamilton ON, Canada. |
| 44 | Tong, Allison | The University of Sydney, Australia. |
| 45 | Morgan-Trimmer, Sarah | School of Primary Care, Population Sciences and Medical Education, University of Southampton, Southampton, SO16 6YD, United Kingdom. |
| 46 | van der Veen, Sabina | Amsterdam UMC, Vrije Universiteit Amsterdam, Department of Ethics, Law and Humanities, de Boelelaan 1118, 1081 HV, Amsterdam, the Netherlands. |
| 47 | van Twillert, Sacha | University of Groningen, University Medical Center Groningen, UMC Staff Policy and Management support, PO Box 30.001, 9700 RB Groningen, the Netherlands. |
| 48 | Varsi, Cecilie | University of South-Eastern Norway, Post office box 4 3199 Borre, Norway. |
| 49 | Verhagen, Evert | Amsterdam Collaboration on Health & Safety in Sports, Department of Public and Occupational Health, Amsterdam Movement Sciences, Amsterdam UMC, University Medical Centres – Vrije Universiteit Amsterdam, Amsterdam, the Netherlands. |
| 50 | Welker, Gera A. | University of Groningen, University Medical Center Groningen, UMC Staff Policy and Management support, PO Box 30.001, 9700 RB Groningen, the Netherlands. |
| 51 | Wensing, Michel | Heidelberg University, Department of General Practice and Health Services Research, INF 130.3, 69120 Heidelberg, Germany. |
| 52 | Wolfenden, Luke | School of Medicine and Public Health, The University of Newcastle, Newcastle, Australia. |
| 53 | Wyss, Kaspar | Swiss Tropical and Public Health Insittute and University of Basel, Kreuzstrasse 2, 4123 Allschwil, Switzerland. |
| 54 | Ziemann, Alexandra | City, University of London, Centre for Healthcare Innovation Research (CHIR), Northampton Square, London EC1V 0HB, United Kingdom. |
| 55 | *1 panel member withdrew from being listed in the group author statement. | |
| Names are listed in alphabetical order. | | |
