# Supplementary material for: CONsensus-based Process evaluation reporting guideline for public HEalth intervention Studies (CONPHES) conducted alongside an effectiveness trial: an e-Delphi study
Source: BMJ Open. 2025 Dec 19;15(12):e093962. doi: 10.1136/bmjopen-2024-093962 (PMC12716498; doi:10.1136/bmjopen-2024-093962)
Supplement: online supplemental file 2 [file bmjopen-15-12-s002.docx]

**Appendix 1. Guideline for Conducting and Reporting of Delphi Studies (CREDES)(18)**

| Rationale for the choice of the Delphi technique | Page |
| --- | --- |
| 1. *Justification.* The choice of the Delphi technique as a method of systematically collating expert consultation and building consensus needs to be well justified. When selecting the method to answer a particular research question, it is important to keep in mind its constructivist nature | 6-7 |
| Planning and design |  |
| 2. *Planning and process.* The Delphi technique is a flexible method and can be adjusted to the respective research aims and purposes. Any modifications should be justified by a rationale and be applied systematically and rigorously | 7-8 |
| 3. *Definition of consensus.* Unless not reasonable due to the explorative nature of the study, an a priori criterion for consensus should be defined. This includes a clear and transparent guide for action on (a) how to proceed with certain items or topics in the next survey round, (b) the required threshold to terminate the Delphi process and (c) procedures to be followed when consensus is (not) reached after one or more iterations | 9 |
| Study conduct |  |
| 4. *Informational input.* All material provided to the expert panel at the outset of the project and throughout the Delphi process should be carefully reviewed and piloted in advance in order to examine the effect on experts’ judgements and to prevent bias | Appendix |
| 5. *Prevention of bias.* Researchers need to take measures to avoid directly or indirectly influencing the experts’ judgements. If one or more members of the research team have a conflict of interest, entrusting an independent researcher with the main coordination of the Delphi study is advisable | Ethical approval and conflicts reported page 15 |
| 6. *Interpretation and processing of results.* Consensus does not necessarily imply the ‘correct’ answer or judgement; (non)consensus and stable disagreement provide informative insights and highlight differences in perspectives concerning the topic in question | 9 |
| 7. *External validation.* It is recommended to have the final draft of the resulting guidance on best practice in palliative care reviewed and approved by an external board or authority before publication and dissemination | 10 |
| Reporting | Page |
| 8. *Purpose and rationale.* The purpose of the study should be clearly defined and demonstrate the appropriateness of the use of the Delphi technique as a method to achieve the research aim. A rationale for the choice of the Delphi technique as the most suitable method needs to be provided | Page 6-7 |
| 9. *Expert panel.* Criteria for the selection of experts and transparent information on recruitment of the expert panel, socio-demographic details including information on expertise regarding the topic in question, (non)response and response rates over the ongoing iterations should be reported | Page 7-8 |
| 10. *Description of the methods.* The methods employed need to be comprehensible; this includes information on preparatory steps (How was available evidence on the topic in question synthesised?), piloting of material and survey instruments, design of the survey instrument(s), the number and design of survey rounds, methods of data analysis, processing and synthesis of experts’ responses to inform the subsequent survey round and methodological decisions taken by the research team throughout the process | Page 7-10 |
| 11. *Procedure.* Flow chart to illustrate the stages of the Delphi process, including a preparatory phase, the actual ‘Delphi rounds’, interim steps of data processing and analysis, and concluding steps | Appendix 3 |
| 12. *Definition and attainment of consensus.* It needs to be comprehensible to the reader how consensus was achieved throughout the process, including strategies to deal with non-consensus | Page 8-9 |
| 13. *Results.* Reporting of results for each round separately is highly advisable in order to make the evolving of consensus over the rounds transparent. This includes figures showing the average group response, changes between rounds, as well as any modifications of the survey instrument such as deletion, addition or modification of survey items based on previous rounds | Page 10-11 and in Appendices |
| 14. *Discussion of limitations.* Reporting should include a critical reflection of potential limitations and their impact of the resulting guidance | Page 4-5 and page 13-14 |
| 15. *Adequacy of conclusions.* The conclusions should adequately reflect the outcomes of the Delphi study with a view to the scope and applicability of the resulting practice guidance | Page 14 |
| 16. *Publication and dissemination.* The resulting guidance on good practice in palliative care should be clearly identifiable from the publication, including recommendations for transfer into practice and implementation. If the publication does not allow for a detailed presentation of either the resulting practice guidance or the methodological features of the applied Delphi technique, or both, reference to a more detailed presentation elsewhere should be made (e.g. availability of the full guidelines from the authors or online; publication of a separate paper reporting on methodological details and particularities of the process (e.g. persistent disagreement and controversy on certain issues)). A dissemination plan should include endorsement of the guidance by professional associations and health care authorities to facilitate implementation | 13 |

**Appendix 2. PubMed search terms**

We have search PubMed (February 2020) and have found 1649 articles. Search terms used were: "process evaluation" [tiab] AND (guide* [tiab] OR standard* [tiab] OR checklist* [tiab] OR framework* [tiab] OR model* [tiab])”.

**Appendix 3: Flow chart of e-Delphi panel members that participated each round**
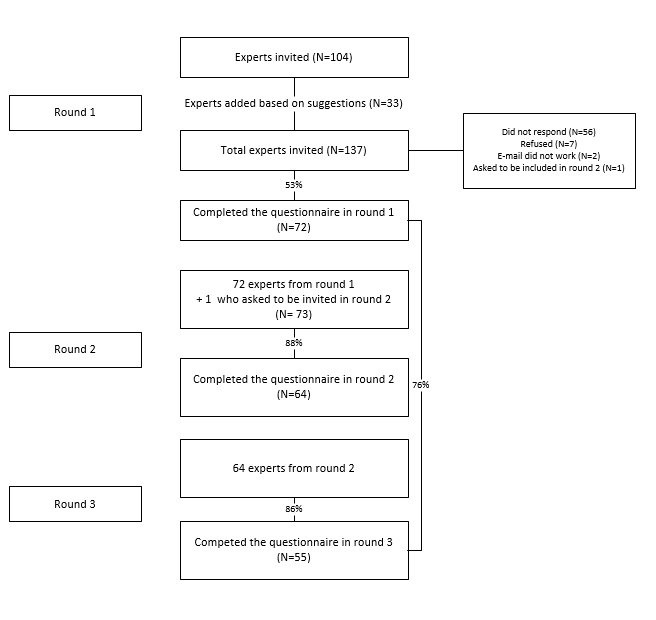


**Appendix 4. Round 1 items, descriptions and consensus**

Overview of the consensus achieved for each item and description of Round 1 of the CONPHES e-Delphi study.

| **Item number** | **Item** |  | **Guide Question/ Description** |  |
| --- | --- | --- | --- | --- |
|  |  | % fully (agree) |  | % fully (agree) |
|  | Domain 1: Intervention and implementation context | 95.6 |  |  |
|  | *Intervention* | 93.1 |  |  |
| 1 | Description of the intervention | 91.7 | Is the intervention described with sufficient details to allow replication?  *Describe the aim of the intervention, components/activities, target population or users, materials for participants, mode of delivery (e.g. face-to-face, online, individual, group-based), duration, frequency, intensity, additional contact time.* | 76.4 |
| 2 | Theoretical underpinnings and mechanisms of the intervention | 90.3 | Are the rationale, theory or mechanisms by which the intervention is expected to work described?  *Describe the elements essential to achieving the desired effect of the intervention in the user.* | 62.0 |
| 3 | Development process | 69.4 | Is the development process of the intervention described?  *Describe materials, research methods or models that were used in the process of developing the intervention, who was consulted or involved in this process, and if the intervention was pilot tested.* | 73.6 |
|  | *Implementation context* | 91.7 |  |  |
| 4 | Context | 88.9 | Is the context in which the intervention is implemented described?  *Describe the organizational setting, enrolment/referral pathways, staff involved, procedural embedding, physical environment, institutional support, political will, required resources, and financial support.* | 72.2 |
| 5 | Delivery setting | 79.2 | Are the type(s) of location(s) or setting(s) where the intervention occurred described?  *Describe, if applicable, eligibility criteria for the location or setting.* | 63.9 |
| 6 | Intervention provider | 80.6 | Is the expertise and background of each category of intervention provider or deliverer described?  *Describe who delivered the intervention.* | 50.0 |
| 7 | Delivery support | 83.3 | Is the support for implementation clearly described?  *Describe implementation strategies, such as materials used for intervention delivery or training of intervention providers or other forms of support.* | 50.0 |
| 8 | Intervention resources | 70.8 | Are the required financial and human resources for intervention delivery described? | 54.2 |
|  | Domain 2: Study design | 93.1 |  |  |
| 9 | Title | 83.3 | Do the title and/or abstract and keywords indicate this is a process evaluation study? | 75.0 |
| 10 | Objective of process evaluation | 94.4 | Are the objective(s) of the process evaluation described? | 80.6 |
| 11 | Recruitment | 87.5 | Is recruitment and selection of participants described?  *Describe, if applicable, incentives for participation, and any eligible criteria set in place.* | 62.5 |
| 12 | Sample size | 76.4 | Are the number of included participants and the demographic characteristics described?  *Describe the sample size calculation, or provide a rational for not conducting a sample size calculation (e.g. budgetary constraints, practical considerations, data saturation).* | 47.2 |
| 13 | Researcher involvement | 72.2 | Is the relationship of the researcher(s) with the study participants (e.g. target population, intervention provider) described?  *Describe how this potentially impacted the intervention delivery and/or outcomes.* | 61.1 |
| 14 | Study setting | 80.6 | Are the type(s) of location(s) where the data was collected described? | 62.5 |
|  | *Data collection* | 95.8 |  |  |
| 15 | Theoretical framework | 76.4 | Is the theory, model or framework that was used to underpin the process outcomes of the intervention described? | 48.6 |
| 16 | Process mechanisms | 62.5 | Is described how process outcomes are related to the mechanisms by which the intervention is expected to work? | 45.8 |
| 17 | Operationalisation process outcomes | 83.3 | Is described how the process outcomes were defined and operationalized into reliable and measurable indicators (i.e. definition provided)?  *Describe, if measured, reach, adoption, recruitment, adherence, fidelity, dosage, quality of delivery, program differentiation, satisfaction, responsiveness, maintenance, context, facilitators and barriers for implementation.* | 52.8 |
| 18 | Data collection methods | 97.2 | Are the methods of data collection described?  *Describe what data sources were used*  *(e.g. questionnaire, interview, focus group, observation, routine collected data, field notes), and what the rationale was for choice of methods.* | 91.7 |
| 19 | Measurement properties | 66.7 | Are measurement properties of the data collection methods described?  *Describe, if applicable, if existing or self-designed methods and instruments were used, and describe their psychometric properties.* | 63.9 |
| 20 | Pilot test methods | 51.4 | Is described if measurement instruments and data collection procedures were pilot tested? | 61.1 |
| 21 | Data collection procedures | 94.4 | Is described how process data was collected?  *Describe the procedures, duration, who collected the data, and who was subject of evaluation.* | 75.0 |
| 22 | Frequency of data collection | 86.1 | Is the timing of data collection and a rationale for the timing described?  *Describe when process data was collected during the trial (i.e. at the start, baseline, during and at the end, repeated measured), and describe the rationale provided for the timing of the data collection.* | 81.9 |
| 23 | Control conditions | 77.8 | Is described, if applicable, if any process data was collected of participants in the control condition? | 69.4 |
|  | Domain 3: Analysis & Findings | 91.7 |  |  |
|  | *Data analysis* | 94.4 |  |  |
| 24 | Timing analysis | 68.1 | Is described if the results of the effectiveness study were known at the time of the analyses?  *Describe the order of the data analyses with regard to the effectiveness evaluation.* | 58.3 |
| 25 | Software | 62.5 | Is software, if applicable, used to analyse the data described? | 63.9 |
| 26 | Analysis | 90.3 | Are (statistical) analyses, if applicable, described?  *Describe the type of analyses and who analyzed the data.* | 66.7 |
| 27 | Techniques to enhance trustworthiness | 90.3 | Are techniques to enhance trustworthiness and credibility of data analysis reported?  *Describe, if applicable, member checking, audit trail, triangulation, and missing data.* | 68.1 |
| 28 | Interaction between process data and effectiveness outcomes | 59.7 | Is the interaction between process evaluation and effectiveness outcomes data described? | 45.8 |
|  | *Reporting* | 83.3 |  |  |
| 29 | Mixed methods | 65.3 | Are quantitative and qualitative data described in an integrated manner (i.e. data triangulation)? | 45.8 |
| 30 | Unintended conditions | 80.6 | Are unintended or unforeseen conditions or factors (e.g. external conditions, side effects, harms) described that occurred during the study which might have influenced the delivery of the intervention? | 58.3 |
| 31 | Representativeness | 68.1 | Is described if the study population and setting were representative for the target population of the intervention beyond the research setting? | 56.9 |
| 32 | Implications | 83.3 | Are implications for policy, practice and/or research beyond the specific research setting discussed? | 83.3 |

Green indicates score of ≥67%, orange indicates score of <67% agreement (20).

**Appendix 5. Round 2 items, descriptions and consensus**

Overview of the consensus achieved for each item and description of Round 2 of the CONPHES e-Delphi study.

| **Item number** | **Item** | **% (strongly) agree** | **Description** | **% (strongly) agree** |
| --- | --- | --- | --- | --- |
| **Title / abstract** | | | |  |
| 1 | Title and abstract | 95.3 | Describe in the title and/or abstract and keywords that the study includes a process evaluation. | * |
| **Introduction** | | | |  |
| 2 | Process evaluation aim(s) and/or objective(s) | 93.8 | Describe aim(s) and/or objective(s) of the process evaluation study. | 85.9 |
| **Methods** | | | |  |
|  | ***Intervention*** |  |  |  |
| 3 | Description of the intervention | * | Describe 1) the intervention, such as the aim of the intervention, components/activities, target population, materials for participants, mode of delivery (e.g. face-to-face, online, individual, group-based), duration and frequency; 2) the intervention development process, such as previous research, frameworks and co-creation that were used in the process of developing the intervention; and 3) if the intervention was pilot tested. *If applicable, refer to other documents reporting on the intervention.* | 81.3 |
| 4 | Context | * | Describe the context in which the intervention is implemented, such as the type(s) of location(s) or setting(s) where the intervention is delivered, physical and geographical environment, socio-cultural and socio-economic context. | 81.3 |
| 5 | Principles and mechanisms of the intervention | 70.3 | Describe the principles and mechanisms by which the intervention is expected to achieve the desired effects. *If applicable, describe the underlying theory and/or logic model.* | 75.0 |
| 6 | Delivery team | 78.1 | If applicable, describe who delivered the intervention and their expertise needed, such as skills, qualifications, and prior experience in delivering the intervention. *If applicable, describe the role of researchers in the delivery of the intervention.* | 76.6 |
| 7 | Implementation strategies | 79.7 | Describe implementation strategies for intervention delivery, such as materials or equipment to support intervention delivery, training of intervention deliverers, audit and feedback, organisational support, and intervention delivery resources. *If applicable, describe the role of researchers in the delivery support.* | 65.6 |
|  | ***Process evaluation design*** |  |  |  |
| 8 | Process evaluation framework | 89.1 | Describe the theory, model or framework (if any) guiding the process evaluation. | 85.9 |
| 9 | Process evaluation outcome(s) | 82.8 | Describe what process evaluation outcome(s) were assessed and how they were defined and operationalised? *If assessed, describe process outcomes, such as reach, adoption, recruitment, fidelity, adherence, dosage (delivered and received), quality of delivery, program differentiation, adaptation, satisfaction, responsiveness, maintenance, costs, context, facilitators and barriers for implementation.* | 78.1 |
|  | ***Data collection*** |  |  |  |
| 10 | Process evaluation recruitment | 87.5 | Describe how, by whom, and when process evaluation participants (e.g. target population, intervention deliverer, location/setting) were recruited, and provide a rationale for the sampling method*. If applicable, describe any eligible criteria set in place and incentives used to increase participant enrolment. If applicable, describe any eligibility criteria for participating location(s) or setting(s).* | 81.3 |
| 11 | Process data collection procedures | 85.9 | Describe 1) what data sources were used (e.g. questionnaire, interview, focus group, observation, routine collected data, field notes); 2) which of the process evaluation outcomes they relate to; 3) the timing of process data collection (e.g. at the study start, during and at the end of the process evaluation study); and 4) data collection setting(s). *If applicable, describe who collected process data, their role in the study, and their relationship with study participants (e.g. target population, intervention deliverer). If applicable, describe if existing or self-designed methods and instruments were used.* | 81.3 |
| 12 | Control conditions | * | Describe if any process data were collected among participants in the control condition. | * |
|  | ***Analysis*** |  |  |  |
| 13 | Process data analysis | 87.5 | Describe 1) the process of data analysis (i.e. qualitative, quantitative and/or mixed methods); 2) who analysed the data; and 3) if the results of the effectiveness study were known at the time of process data analysis. *If applicable, name and describe the software used to facilitate the analysis. If applicable, describe how data integration techniques were conducted to integrate quantitative and qualitative data.* | 76.6 |
| 14 | Quality assurance of data analysis | 85.9 | Describe what techniques were used to ensure the quality of qualitative and/or quantitative data analysis? *If applicable, describe processes such as member checking, audit trail, triangulation, data cleaning, blinding, and how missing data were handled.* | 87.5 |
| **Results** | | | |  |
| 15 | Demographic characteristics of process evaluation participants | 78.1 | Describe demographic characteristics of participants included in the process evaluation (e.g. target population, intervention deliverer, setting), and describe if the process evaluation study population and setting(s) were representative for the target population of the effectiveness trial. *If assessed, describe demographic characteristics of the control or comparison condition participants.* | 75.0 |
| 16 | Process evaluation outcome(s) | 65.6 | Describe all process evaluation outcome(s) that were assessed.  *If applicable, describe the relationship between process evaluation findings and effectiveness outcomes (i.e. dose-response analysis).* | 62.5 |
| 17 | Influential factors | 54.7 | Describe (unexpected) factors which may have influenced the implementation of the intervention during the effectiveness study, such as individual, organisational, contextual, and/or political factors. | 56.3 |
| **Discussion** | | | |  |
| 18 | Interpretation of findings | 79.7 | Describe how effectiveness outcomes should be interpreted in light of the process evaluation findings. | 71.9 |
| 19 | Implications | * | Discuss implications of the process evaluation outcomes for the intervention and its delivery beyond the specific research setting, and implications for policy, practice and/or research. | 89.1 |

Green indicates score of ≥67%, orange indicates score of <67% agreement (20) | * already reached consensus in the previous round.

**Appendix 6. Round 3 items, descriptions and consensus**

Overview of the consensus achieved for each item and description of Round 3 of the CONPHES e-Delphi study.

| **Item number** | **Item** | **% (strongly) agree** | **Description** | **% (strongly) agree** |
| --- | --- | --- | --- | --- |
| **Title / abstract** | | | |  |
| 1 | Title and abstract | * | Describe in the title and/or abstract and keywords that the study includes a process evaluation. | * |
| **Introduction** | | | |  |
| 2 | Process evaluation aim(s) and/or objective(s) | * | Describe aim(s) and/or objective(s) of the process evaluation study. | * |
| **Methods** | | | |  |
|  | ***Intervention*** |  |  |  |
| 3 | Description of the intervention | * | Describe 1) the intervention development process, with reference to e.g. previous research, frameworks and co-creation that were used in the process of developing the intervention; 2) the intervention, such as the aim of the intervention, components/activities, target population, materials for participants, mode of delivery (e.g. face-to-face, online, individual, group-based), duration and frequency; and 3) if the intervention was pilot tested. *If applicable, refer to other documents that describe the intervention.* | * |
| 4 | Implementation strategies | * | Describe implementation strategies (methods or techniques) used to enhance the adoption, implementation and sustainability of the intervention such as materials to support intervention delivery, training of intervention deliverers, organisational support or intervention delivery resources.  *If applicable, refer to other documents that describe the implementation strategies.* | 90.8 |
| 5 | Mechanisms of the intervention | * | Describe the mechanisms by which the intervention and implementation strategies are expected to achieve the desired effects. *If applicable, present a theory of change or logic model.* | * |
| 6 | Delivery and support team | * | If applicable, describe who delivered the intervention and implementation strategies, and their expertise such as skills, qualifications, and prior experience in delivering the intervention or implementation strategies. *If applicable, describe any potential role members of the research team had.* | * |
| 7 | Context | * | Describe the context in which the intervention was implemented, such as the type(s) of location(s) or setting(s) where the intervention was delivered, the physical and geographical environment(s), and the socio-cultural and socio-economic context(s). | * |
|  | ***Process evaluation design*** |  |  |  |
| 8 | Process evaluation framework | * | Describe the theory, model or framework guiding the process evaluation. | * |
| 9 | Process evaluation outcome(s) | * | Describe process evaluation outcome(s) that were assessed and how they were defined and operationalised. *If assessed, describe process evaluation outcomes such as reach, adoption, recruitment, fidelity, adherence, dosage (delivered and received), quality of delivery, program differentiation, adaptation, satisfaction, responsiveness, maintenance, costs, facilitators and barriers for implementation.* | * |
|  | ***Data collection*** |  |  |  |
| 10 | Recruitment for the process evaluation | * | Describe how, by whom, and when process evaluation participants (e.g. target population, intervention deliverer, location/setting) were recruited, and provide a rationale for the recruitment strategy*. If applicable, describe any eligibility criteria and/or incentives used for participant enrolment. If applicable, describe any eligibility criteria for the location(s) or setting(s).* | * |
| 11 | Process evaluation data collection procedures | * | Describe 1) what data sources were used (e.g. questionnaire, interview, focus group, observation, routine collected data, field notes); 2) which of the process evaluation outcomes they relate to; 3) the timing of process data collection (e.g. at the study start, during and at the end of the process evaluation study); and 4) data collection setting(s). *If applicable, describe who collected process data, their role in the study, and their relationship with study participants (e.g. target population, intervention deliverer). If applicable, describe whether pre-existing methods and instruments were used or whether they were developed specifically for this study.* | * |
| 12 | Control group data collection | * | Describe if any process evaluation data were collected among participants in the control or comparison group(s). | * |
|  | ***Analysis*** |  |  |  |
| 13 | Process evaluation data analysis | * | Describe 1) data analysis (i.e. qualitative, quantitative and/or mixed methods); 2) who analysed the data; and 3) if the results of the effectiveness study were known at the time of process data analysis. *If applicable, name and describe the software used to facilitate the analysis. If applicable, describe how data integration techniques were conducted to integrate quantitative and qualitative data.*  *If applicable, describe if the relationship between process evaluation findings and effectiveness outcomes were assessed (e.g. dose-response analysis).* | * |
| 14 | Quality assurance of data analysis | * | Describe techniques that were used to ensure the quality of qualitative and/or quantitative data analysis. *If applicable, for qualitative analyses describe processes such as member checking, audit trail, and triangulation. For quantitative analyses describe processes such as data cleaning, blinding, and how missing data were handled.* | * |
| **Results** | | | |  |
| 15 | [Characteristics of process evaluation participants](#_comments_and_suggestion_9) | * | Describe characteristics of participants in the process evaluation, and, where relevant, the intervention deliverer(s) and setting(s), and describe to what degree the process evaluation participants and setting(s) were representative of the target population of the effectiveness trial. *If assessed, describe characteristics of the control or comparison group participants.* | * |
| 16 | Results of process evaluation | 85.5 | Describe the results of the process evaluation as defined in the methods section (as listed in items 9 and 13). | 80.0 |
| **Discussion** | | | |  |
| 17 | Interpretation of findings | * | Describe how the findings of the process evaluation can be interpreted.  *If applicable, describe how effectiveness outcomes should be interpreted in light of the process evaluation findings.* | * |
| 18 | Unexpected factors | 67.3 | Describe unexpected factors that may have influenced the implementation of the intervention during the effectiveness study. | 60.0 |
| 19 | Implications | * | Discuss implications of the process evaluation outcomes for future intervention delivery, and implications for policy, practice and/or research. | * |

Green indicates score of ≥67%, orange indicates score of <67% agreement (20) | * already reached consensus in the previous rounds.

**Appendix 7. Checklist for completion by authors submitting a process evaluation paper**

| **Item number** | **Item** | **Description** | **Reported on Page No.** |
| --- | --- | --- | --- |
| **Title/Abstract** | | | |
| 1 | Title and abstract | Describe in the title and/or abstract and keywords that the study includes a process evaluation. |  |
| **Introduction** | | | |
| 2 | Process evaluation aim(s) and/or objective(s) | Describe aim(s) and/or objective(s) of the process evaluation study. |  |
| **Methods** | | | |
| ***Intervention*** | | | |
| 3 | Description of the intervention | Describe 1) the intervention development process, with reference to e.g. previous research, frameworks and co-creation that were used in the process of developing the intervention; 2) the intervention, such as the aim of the intervention, components/activities, target population, materials for participants, mode of delivery (e.g. face-to-face, online, individual, group-based), duration and frequency; and 3) if the intervention was pilot tested. *If applicable, refer to other documents that describe the intervention.* |  |
| 4 | Implementation strategies | Describe implementation strategies (methods or techniques) used to enhance the adoption, implementation and sustainability of the intervention such as materials to support intervention delivery, training of intervention deliverers, organisational support or intervention delivery resources.  *If applicable, refer to other documents that describe the implementation strategies.* |  |
| 5 | Mechanisms of the intervention | Describe the mechanisms by which the intervention and implementation strategies are expected to achieve the desired effects. *If applicable, present a theory of change or logic model.* |  |
| 6 | Delivery and support team | If applicable, describe who delivered the intervention and implementation strategies, and their expertise such as skills, qualifications, and prior experience in delivering the intervention or implementation strategies. *If applicable, describe any potential role members of the research team had.* |  |
| 7 | Context | Describe the context in which the intervention was implemented, such as the type(s) of location(s) or setting(s) where the intervention was delivered, the physical and geographical environment(s), and the socio-cultural and socio-economic context(s). |  |
| ***Process evaluation design*** | | | |
| 8 | Process evaluation framework | Describe the theory, model or framework guiding the process evaluation. |  |
| 9 | Process evaluation outcome(s) | Describe outcome(s) that were assessed in the process evaluation and how they were defined and operationalised. *If assessed, describe process evaluation outcomes such as reach, adoption, recruitment, fidelity, adherence, dosage (delivered and received), quality of delivery, program differentiation, adaptation, satisfaction, responsiveness, maintenance, costs, facilitators and barriers for implementation.* |  |
| ***Data collection*** | | | |
| 10 | Recruitment for the process evaluation | Describe how, by whom, and when process evaluation participants (e.g. target population, intervention deliverer, location/setting) were recruited, and provide a rationale for the recruitment strategy*. If applicable, describe any eligibility criteria and/or incentives used for participant enrolment. If applicable, describe any eligibility criteria for the location(s) or setting(s).* |  |
| 11 | Process evaluation data collection procedures | Describe 1) what data sources were used (e.g. questionnaire, interview, focus group, observation, routine collected data, field notes); 2) which of the process evaluation outcomes they relate to; 3) the timing of process data collection (e.g. at the study start, during and at the end of the process evaluation study); and 4) data collection setting(s). *If applicable, describe who collected process data, their role in the study, and their relationship with study participants (e.g. target population, intervention deliverer). If applicable, describe whether pre-existing methods and instruments were used or whether they were developed specifically for this study.* |  |
| 12 | Control group data collection | Describe if any process evaluation data were collected among participants in the control or comparison group(s). |  |
| ***Analyses*** | | | |
| 13 | Process evaluation data analysis | Describe 1) data analysis (i.e. qualitative, quantitative and/or mixed methods); 2) who analysed the data; and 3) if the results of the effectiveness study were known at the time of process data analysis. *If applicable, name and describe the software used to facilitate the analysis. If applicable, describe how data integration techniques were conducted to integrate quantitative and qualitative data.*  *If applicable, describe if the relationship between process evaluation findings and effectiveness outcomes were assessed (e.g. dose-response analysis).* |  |
| 14 | Quality assurance of data analysis | Describe techniques that were used to ensure the quality of qualitative and/or quantitative data analysis. *If applicable, for qualitative analyses describe processes such as member checking, audit trail, and triangulation. For quantitative analyses describe processes such as data cleaning, blinding, and how missing data were handled.* |  |
| **Results** | | | |
| 15 | Characteristics of process evaluation participants | Describe characteristics of participants in the process evaluation, and, where relevant, the intervention deliverer(s) and setting(s), and describe to what degree the process evaluation participants and setting(s) were representative of the target population of the effectiveness trial. *If assessed, describe characteristics of the control or comparison group participants.* |  |
| 16 | Results of process evaluation | Describe the results of the process evaluation as defined in the methods section (as listed in items 9 and 13). |  |
| **Discussion** | | | |
| 17 | Interpretation of findings | Describe how the findings of the process evaluation can be interpreted.  *If applicable, describe how effectiveness outcomes should be interpreted in light of the process evaluation findings.* |  |
| 18 | Unexpected factors | Describe previous unknown factors that may have influenced the implementation of the intervention and/ or the process evaluation results. |  |
| 19 | Implications | Discuss implications of the process evaluation outcomes for future intervention delivery, and implications for policy, practice and/or research. |  |

**Appendix 8: Exploration and Elaboration (E&E) document**

A process evaluation is a type of research that aims to assess how well an intervention or program is implemented and delivered. It focuses on the implementation process rather than the effectiveness of the intervention itself. This reporting guideline helps you to report your research in a clear and transparent manner, and help you to ensure that relevant information is included in your manuscript. We do acknowledge that extensively reporting on all details of items might not be feasible due to word limits of journals. However, we suggest to include such relevant information in an appendix. Furthermore, you could possibly also refer to other documents that describe the intervention, such as study protocol papers, design papers, or an effectiveness paper.

Although we ordered the items according to the common structure of a manuscript (i.e. introduction-methods-results-discussion), we leave it up to the authors to decide the order in which they describe the individual items.

This document is structured in a uniform matter, with each item being identified by a number (ranging from 1 to 19). Following this, the reporting approach for the item is illustrated through examples (drawn from published articles or fictional) and corresponding explanations.

| **Item number** | **Item** | **Description** |
| --- | --- | --- |
| 1 | Title and abstract | Describe in the title and/or abstract and keywords that the study includes a process evaluation. |
| **Example:**  *Title:* ***Process evaluation*** *of workplace health promotion in a sheltered workplace: a care ethics perspective (21)*  *Abstract:* *A responsive* **process evaluation** *of an extensive multi-component workplace health promotion program targeting lifestyle behaviors, financial behaviors, literacy and citizenship, was performed in a large, sheltered workplace in the Netherlands (>3500 employees).*  **Explanation:** It is important to include the process evaluations study design in the title and/ or abstract. This serves the purpose of enabling readers to easily identify the study as a process evaluation conducted alongside an effectiveness trial of public health interventions, and also helps to ensure correct indexing of the article in electronic databases. It is worth noting that some journals may not allow the use of keywords that are similar to those used in the title, therefore it may be useful to include the term ‘process evaluation’ in other parts of the manuscript or use synonyms such as implementation evaluation, hybrid trial, formative evaluation, program monitoring or program evaluation as key words. | | |
| 2 | Process evaluation aim(s) and/or objective(s) | Describe aim(s) and/or objective(s) of the process evaluation study. |
| **Example:** *The overall aim of this study was to evaluate the implementation process of the Dynamic Work intervention. The objectives of this study were 1) to investigate the context, implementation, and mechanism of impact of the Dynamic Work intervention following the UK Medical Research Council (MRC) guidance for process evaluations (1) (Moore et al., 2015), and 2) to explore the association between the degree of implementation and changes in participants’ outcomes.*  **Explanation:** Including a description of the aim(s) and/or objective(s) can help to clearly outline what the researcher’s intentions are with the study. While aims and objectives are difficult to differentiate, aims generally refer to the question that the process evaluation was designed to answer, and are often referred to as research question and purposes. Research objectives, on the other hand, are specific, measurable, and time bound statements that describe the steps that a researcher will take to achieve the research aim. Objectives are most of the time more specific than the aim. It is important not to mix the aims and/or objectives of the process evaluation with the aims/objectives of the effectiveness study. Furthermore, aims/objectives can be formulated not only for the intervention that is being evaluated in the process evaluation, but also for the implementation strategies that are being evaluated (as discussed in item 4). | | |
| 3 | Description of the intervention | Describe 1) the intervention development process, with reference to e.g. previous research, frameworks and co-creation that were used in the process of developing the intervention; 2) the intervention, such as the aim of the intervention, components/activities, target population, materials for participants, mode of delivery (e.g. face-to-face, online, individual, group-based), duration and frequency; and 3) if the intervention was pilot tested. *If applicable, refer to other documents that describe the intervention.* |
| **Example:** *MindTheGap is an evidence-based online app that aims to help employees experiencing high levels of stress to transition well into a holiday period. MindTheGap is based on principles of mindfulness training (breathing techniques and walking meditation) and cognitive behavioural therapy (psychoeducation and behavioural activation). MindTheGap is a self-help tool that is delivered online through an interactive website suitable for both large (PC’s laptop, tablets) and small screens (smart phones). Participants will go through a three-day interactive journal which guides them through 30-60 minutes of reading, relaxing and activation activities each day. The various underlying therapeutic principles are research based and tested for efficacy and effectiveness for different populations. The user interface, design and logical structure of MindTheGap has been developed in three interactive workshops with employees (n=15) at two different companies in the financial and medical sector. The logical model and description of the active components and the essentials of the technical implementation of MindTheGap are described in Annex X.*  **Explanation:** This item describes an important part of the ‘*what’* is being evaluated in the process evaluation: the intervention and the process by which it has been developed. For example by methods using co-creation of the target population and/ or stakeholders, or the use of a framework such as the Intervention Mapping protocol (22) or the Behavior Change Wheel (23). This can help to assess the intervention’s robustness and the strength of the evidence supporting it. To ensure the results of the process evaluation can be properly interpreted, it is crucial to provide a substantial level of detail of the intervention, such as the aim of the intervention, components/activities, target population (i.e. who the intervention was developed for), materials for participants, mode of delivery (e.g. face-to-face, online, individual, group-based), duration and frequency.  It is encouraged to use an established standard method to describe the intervention, such as the TiDIER checklist (24) describing: why, what, who provides, how, where, when, how much, how well, and modifications. It is also important to explain the process by which the intervention was piloted and how this influenced the development process and design of the intervention that was evaluated in the process evaluation.  Finally, if possible, refer to other documents that describe the intervention, such as study protocol papers, design papers, or an effectiveness paper. | | |
| 4 | Implementation strategies | Describe implementation strategies (methods or techniques) used to enhance the adoption, implementation and sustainability of the intervention such as materials to support intervention delivery, training of intervention deliverers, organisational support or intervention delivery resources.  *If applicable, refer to other documents that describe the implementation strategies.* |
| **Example:** *All involved three primary care and two community health care organisations were committed to implement the intervention. For the primary care practices a blended strategy consisting of two discrete strategies addressing two different target groups was used. This consisted of a brief 2-hour technical training for the practitioner support staff and a paper-based self-screener for patients in the waiting room. This screener could then be used to prep the meeting with their GP. For implementing the intervention in community settings, a similar implementation strategy was followed. The training was adapted to the particular support practitioner (mental health, physiotherapy, diet, physical exercise, etc.). In addition, the screener was delivered digitally through a table to optimise information flow and increase efficiency. The implementation strategies were developed using the CFIR-ERIC strategy matching tool (ref) and operationalised with a small working group with representatives from both target groups (patients and professionals). In three workshops, the working group evaluated options and pilot tested by using role playing for working processes of the implementation strategies. The most important evaluation criteria used in this included usability and increased diagnostic precision. The description of each implementation strategy component is included in Annex X.*  **Explanation:** This item focuses on implementation strategies: “methods or techniques used to enhance the adoption, implementation, and sustainability of a program or practice”, also known as the “*how to*” of implementation (25, 26). Adoption refers to the initial decision- and decision-making process to take up the intervention that precedes the (deliberate and planned) implementation (27). Implementation in turn, concerns the planned and deliberate process of integrating and embedding a new intervention into an existing practice (27, 28). Sustainability relates to the activities required to maintain the implemented intervention as a normal part of practice.  Implementation strategies may be discrete, multifaceted or blended and can consist of singular activities addressing a singular barrier or multiple actions addressing multiple barriers at various levels (29). Similar to the relevance of describing the intervention that is implemented (item 3), it is also crucial to provide a detailed description of the implementation methods to facilitate interpretation of the process evaluation findings. When describing the implementation strategies, it might be helpful to use the reporting standard suggested by Proctor and colleagues (25). This standard requires providing a name for the implementation strategy, a link to existing taxonomies of strategies, and an operationalization of the actor, the action, the target, timelines, dose, outcomes, and requirements, as well as any empirical, theoretical or pragmatic justification of the choice of the strategy.  Finally, if possible, refer to other documents that describe the intervention, such as study protocol papers, design papers, or an effectiveness paper. | | |
| 5 | Mechanisms of the intervention | Describe the mechanisms by which the intervention and implementation strategies are expected to achieve the desired effects. *If applicable, present a theory of change or logic model or refer to other documents that describe the mechanisms of the intervention.* |
| **Example:** *Figure 1 depicts the logic model of the program. The content of the logic model – the theory of how EuroFIT affects outcomes and how the program is implemented in the context of the football club – will be examined through 18 research objectives (ROs)(30).*  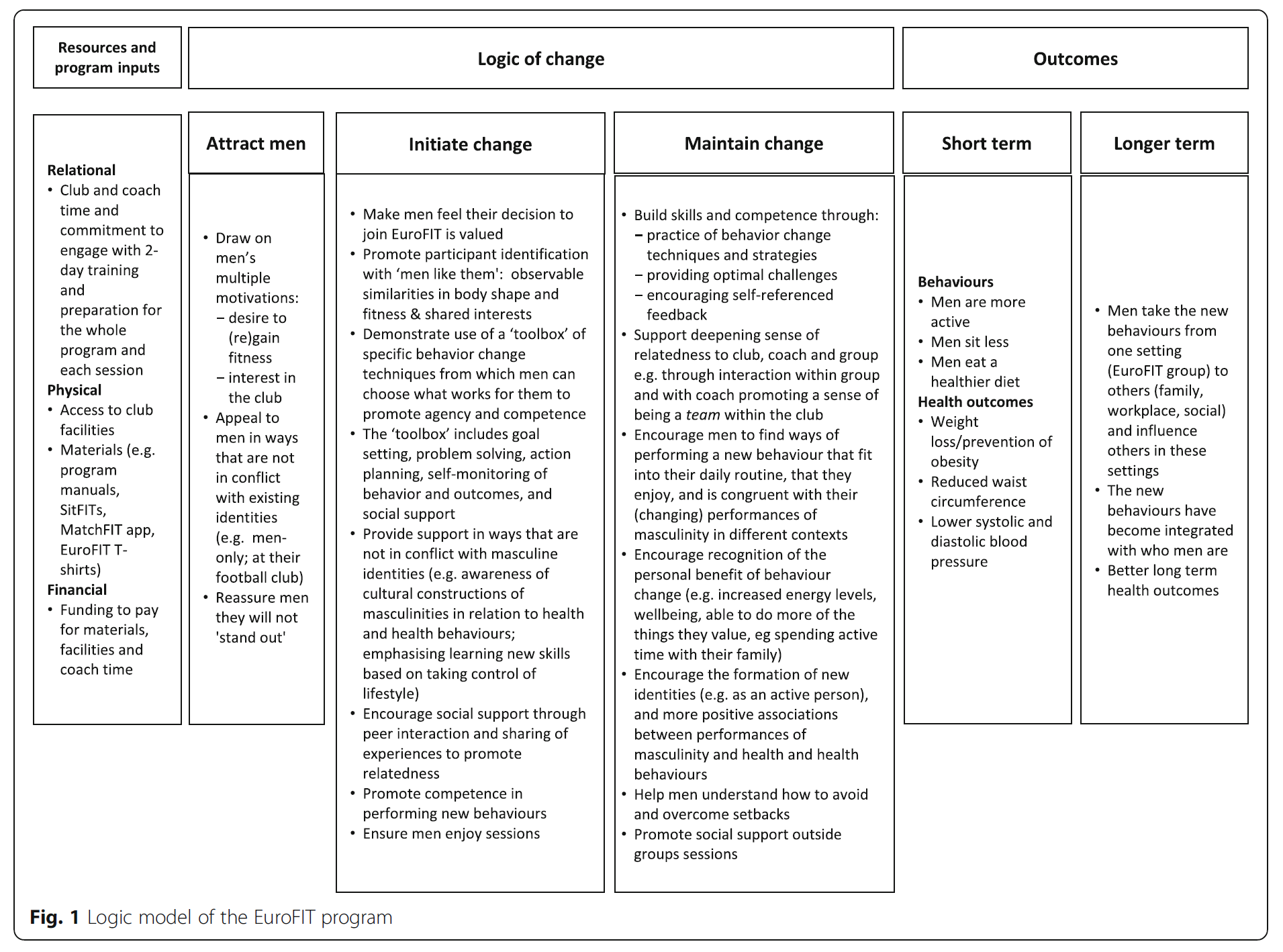  *The implementation strategy was developed iteratively using an Agile Science approach as proposed by Lewis and colleagues (31). Starting from the objectives and expected outcomes formulated in consultation with healthy volunteers in the group with relative high risk of developing cancer, we developed the following causal pathway for our implementation strategy.*  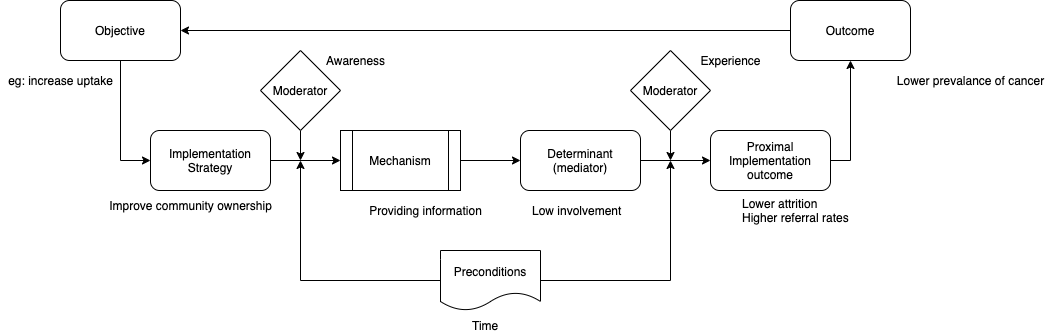  **Explanation:** It is essential to understand the mechanisms by which an intervention leads to change in order to interpret the effects of the specific intervention and how these effects might be replicated by similar interventions. This is applicable to both the intervention that is implemented and the strategy used to implement it.  To accomplish this, a logic model or theory of change should be provided, outlining the casual pathways through which the intervention and/or implementation strategy(ies) achieve its intended outcome. The model should include a brief description of the intervention objectives, required inputs, processes and actions (i.e. mechanisms), potential moderating and mediating factors, preconditions, and expected (proximal) outcomes. A figure can be helpful. The logic model should be explaining causality with or without identification of a specific theory, theoretical framework, or model. It is important to clearly indicate the evidence-base of the logic model of the intervention and implementation strategy and what is hypothesis-based. By developing such a logic model, it is possible to use the process evaluation to test and refine the logic model.  Finally, if possible, refer to other documents that describe the intervention, such as study protocol papers, design papers, or an effectiveness paper. | | |
| 6 | Delivery and support team | If applicable, describe who delivered the intervention and implementation strategies, and their expertise such as skills, qualifications, and prior experience in delivering the intervention or implementation strategies. *If applicable, describe the role(s) members of the research team had.* |
| **Example:** *The intervention was delivered by specialised nurses. They were either required to be certified acute care nurse practitioner or in training and being supervised by a certified nurse. Nurses should have demonstrated experience with pain management. The implementation was supported by two (on site) project managers who were also employed by the project and part of the research team. The project managers were responsible for recruitment of nurses, organising the on-site training and monitoring fidelity to the intervention guidelines. Both project managers had a background in research and were previously involved in rolling out digitalisation of the patient health records in their respective hospitals.*  **Explanation:** Most, if not all, of the work in this context involves human beings. To understand and replicate the outcomes achieved, it is important to understand who was involved and in what capacity. Information on the characteristics, skills, expertise, experience, training received, and roles and responsibilities of the people involved can aid in defining the minimal requirements for the delivery (i.e. those who deliver the intervention to the target population) and the support team (i.e. those who support the deliverers by applying implementation strategies). Some of these elements might be covered in items 3 and 4, while item 6 complements these items by describing their characteristics, roles and responsibilities. This concerns (only) persons who were directly involved in the delivery of the intervention and persons directly involved in delivery of the implementation strategy or strategies. This information is especially important to inform implementation beyond the research context, i.e. have insight in the roles that are often executed by the research team as part of the trial, but are actually an implementation strategy (see item 4). | | |
| 7 | Context | Describe the context in which the intervention was implemented, such as the type(s) of location(s) or setting(s) where the intervention was delivered, the physical and geographical environment(s), and the socio-cultural and socio-economic context(s). |
| **Example:** *The implementation study took place in the capital of region x. The 12 participating gyms and activity centers were located in each district serving a total of 12,000 persons a year with physical activity programs. The participating organisations differed in size, from small scale charity-based activity centers in residential areas to two large gyms in the business center. Each participating organization had at least one person appointed to guide the implementation. None of the organisations received funding from the project but were funded through various means, including through subscriptions, event-based activities, and gifts.*  **Explanation:** Context is a multifaceted term incorporating various levels and aspects of the system (32). Context encompasses determinants that are pre-existing, dynamic, intertwined and emerge throughout the implementation process. When implementing complex interventions in practice settings, the impact of the combined components on the implementation process is greater that individual factors. Therefore, it is crucial to consider the context when reporting the findings of a process evaluation.  Acknowledging that implementation takes place in a context and that is impacted by the configuration of local services and variation in attitudes, norms, skills, etc. of those expected to change (33) and those who make the change happen, makes it thus important to accurately describe the context when reporting findings of a process evaluation. The context refers to everything else that is not the intervention, including the determinants of implementation that are independent of the intervention.  Following an extensive review on defining context in relation to implementation (32), a description could include the following elements: individual factors (e.g. perceptions/ attitudes, levels of autonomy, or self-efficacy), team-based factors (teamwork), organisational factors (basic characteristics, resources, culture, climate), external contextual factors (economic, political, social), and/or multi-level contextual factors (resources, leadership, culture, evaluation, social capital, implementation setting). | | |
| 8 | Process evaluation - framework | Describe the theory, model or framework guiding the process evaluation. |
| **Example:** *In drafting the study protocol we followed the guideline for process evaluations of complex interventions by the UK Medical Research Council (MRC) (1). We used the MRC framework to structure the sub-research questions in three domains: context, implementation, and impact. In each of these domains, different data sources were identified including systematic reviews (notably for contextual factors), interviews, observations and validated questionnaires (to assess the implementation process and outcomes). In addition, we used a Realist Evaluation (34) approach to develop Context-Mechanism-Outcome (CMO) statements to inform the mechanisms of impact.* *In establishing the CMO-statements and defining the data sources and methods, we consulted periodically with the intervention owners and target users. We established regular update and Q&A meetings with the same group who will continue during the study to discuss progress, clarify issues and emerging findings, and decide collectively on solutions to ensure good quality working relations.*  **Explanation:** Specify the theory, model or framework that guided the design and execution of the process evaluation. This can include a single theory, model or framework, or a combination of different approaches, and the rational for using a specific or combination should be clearly justified. Furthermore, it is important to describe how the chosen framework has influenced the evaluation design, the selection of methods, and the analytical framework used for data analysis. Per Nilsen (35) has described different theories, models and frameworks that can be used. Common used ones are: RE-AIM (36), Proctor implementation outcomes (27), UK MRC guidelines for process evaluations (1), Realist Evaluation (2), Linnan and Steckler (2), Durlak and Dupre (5), Normalisation Process Theory (37). | | |
| 9 | Process evaluation - outcome(s) | Describe any outcome(s) that were assessed in the process evaluation and how they were defined and operationalised. *If assessed, describe process evaluation outcomes such as reach, adoption, recruitment, fidelity, adherence, dosage (delivered and received), quality of delivery, program differentiation, adaptation, satisfaction, responsiveness, maintenance, costs, facilitators and barriers for implementation.* |
| **Example:** *The process evaluation is part of a hybrid (type 2) implementation study consisting of an RCT on cost-effectiveness of the steppedR2W-app in a public service setting. We will start our process evaluation by describing what has been delivered in the participating municipalities in practice. We will collect quantitative and organisational data on uptake and usage (logfiles), as well as a user survey for their perceived usability of the app using the SUS questionnaire. Usage data will be collected at three time points to detect possible change over time. In our interviews and document analysis we will include both usage data and acceptability information to identify patterns (e.g. departmental structure, age, gender, etc.) to develop plausible reasons why certain patterns have occurred in the usage or acceptability data.*  **Explanation:** Process evaluation outcomes refer to the impacts that result from the implementation and/or delivery process of an intervention. The explanation of item 9 provides some examples of process evaluation outcomes such as reach, adoption, recruitment, fidelity, adherence, dosage (delivered and received), quality of delivery, program differentiation, adaptation, satisfaction, responsiveness, maintenance, costs, and facilitators and barriers for implementation. These outcomes can be derived from an evaluation framework, such as the RE-AIM model (3) Saunders (38), Proctor implementation outcomes (27), UK MRC guidelines for process evaluations (1), Linnan and Steckler (2), or Durlak and Dupre (5). Within the guideline, we do not aim to provide guidance on what outcomes to select for a study, nor do we want to provide an exhaustive list of outcomes. Yet, we do aim to inform users of the guideline on what type of outcomes might be relevant in a process evaluation study, and might therefore be relevant to report in a manuscript.  It is important to specify what kind of outcomes are included, why they are important, and how they will inform the evaluation and complement other data sources and methods used in the process evaluation. Additionally, it is important to describe the relationship and rationale with the effectiveness outcomes (as outlined in a logic model – see item 5). | | |
| 10 | Recruitment for the process evaluation | Describe how, by whom, and when process evaluation participants (e.g. target population, intervention deliverer, location/setting) were recruited, and provide a rationale for the recruitment strategy*. If applicable, describe any eligibility criteria and/or incentives used for participant enrolment. If applicable, describe any eligibility criteria for the location(s) or setting(s).* |
| **Example:** *For the interviews we recruited primary care nurse practitioners working in General Practitioner offices in region x and y. All General Practitioners registered at the municipality were contacted by email asking to nominate and consent to contact one or more nurses at their practice. The nurses were contacted by email informing them about the study with a standardised information leaflet and consent form asking them if they would be willing to participate in the study. When they confirmed, we contacted them by phone and made sure the login was working. Recruitment was done by authors AB and BE in Spring 2018. Only primary care nurses with a psychiatric mental health certification were eligible to participate.*  **Explanation**: A process evaluation should result in systematic, verifiable and, whenever possible, generalisable knowledge about how certain outcomes were achieved. The sample that is being studied should therefore be clearly defined and described in terms of general relevant characteristics such as age and experience. A rationale why especially these participants were recruited can be helpful (i.e. targeting all participant, those performing well, those not/very satisfied, etc.). Additionally, the recruitment strategy and methods should be thoroughly explained, including the use of information materials, how potentially eligible participants were contacted, and the criteria used to include or exclude participants or groups of participants for your process evaluation. If participants were nested within organizations, it is important to describe how the organizations were included and whether any incentives were offered to encourage participation. Be aware that the recruitment for a process evaluation participant might vary from those participating in the effectiveness trial, as often only a sub-sample will be partaking in the process evaluation (i.e. often the intervention arm of an effectiveness trial). | | |
| 11 | Process evaluation data collection procedures | Describe 1) what data sources were used (e.g. questionnaire, interview, focus group, observation, routine collected data, field notes); 2) which of the process evaluation outcomes they relate to; 3) the timing of process data collection (e.g. at the study start, during and at the end of the process evaluation study); and 4) data collection setting(s). *If applicable, describe who collected process data, their role in the study, and their relationship with study participants (e.g. target population, intervention deliverer). If applicable, describe whether pre-existing methods and instruments were used or whether they were developed specifically for this study.* |
| **Example:** *Quantitative data were collected through participant questionnaires in the intervention arm of the trial (n = 500), coach questionnaires (n = 30), attendance sheets and coach logs of sessions delivered (n = 360) and participants’ logs from the SitFIT and the game-based app MatchFIT. Observations of intervention sessions, interviews and focus group discussions with participant, coaches and club representatives were conducted (30).*  *The research team in each country was responsible for securing collaboration with local partners, recruitment of the clubs, implementation support for the clubs, and data sampling during the trial (39).*  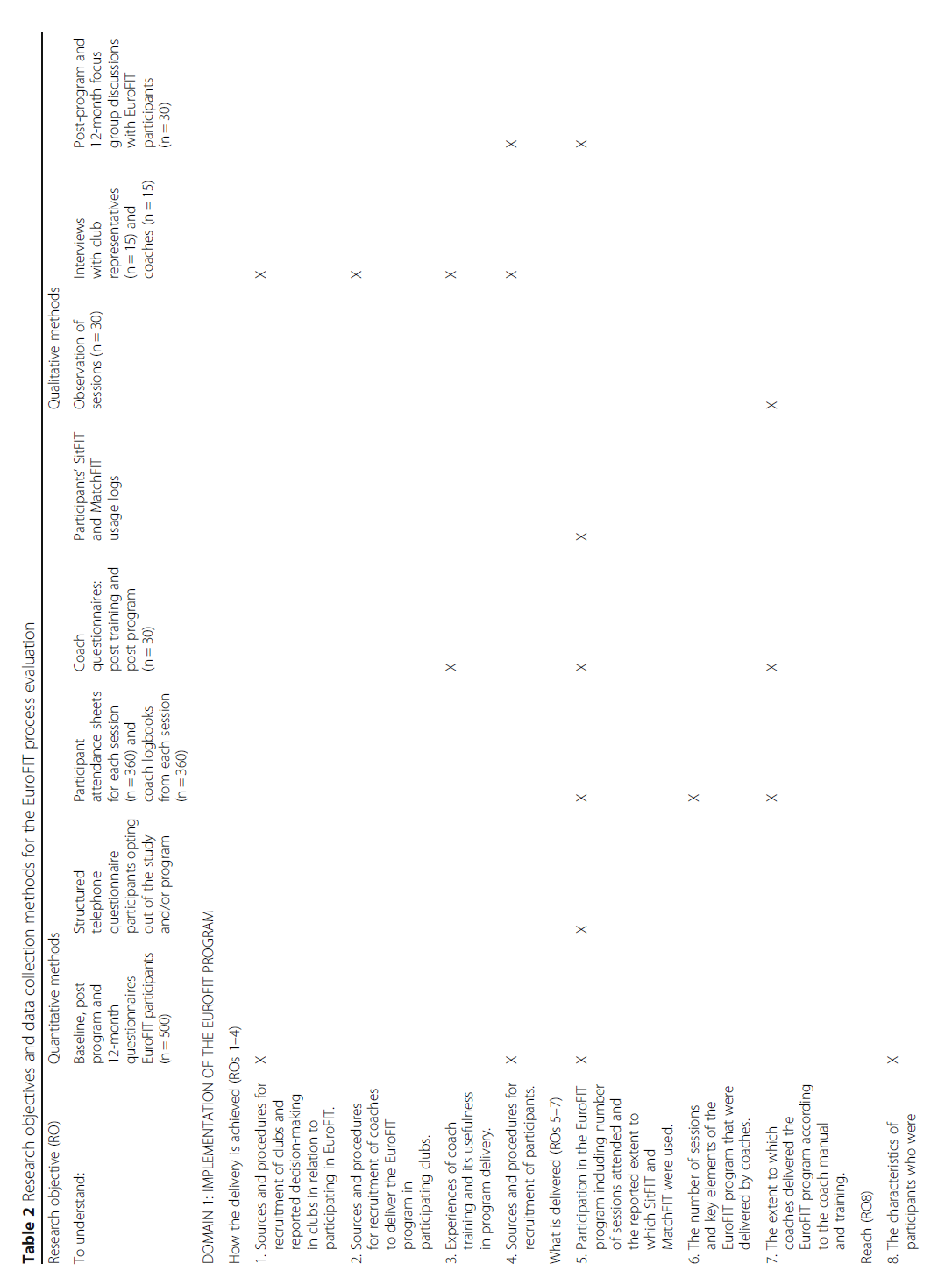  **Explanation:** The methodology of a process evaluation should be designed based on the research question, and the methods should be chosen accordingly. A methodology is generally understood as the overall strategy that organises the research process, including methods. A method is generally regarded as a (set of) data collection procedure(s) and analysis tool(s). Information on the type of data (i.e. quantitative, qualitative or mixed methods) is important. With regard to listing the chosen methods and instruments, it will be helpful to report whether the instruments were self-developed or if an existing instrument was used. Reference to manuscripts reporting their previous use will be helpful, as well as adding the instruments (i.e. questionnaire, interview topic guide etc.) as an appendix to the manuscript. Sometimes the choice of a data collection method is ambivalent. It is therefore important to be transparent about the reasons for choosing a certain method and to describe its potential strengths and limitations.  Furthermore, data is time dependent. Therefore, information on the order and timing of data collection methods (i.e. pre-implementation, during implementation, during training, after implementation) can be helpful to establish whether a certain claim is rightfully based on the collected data. It might be helpful to draft a matrix to outline how each data collection method is related to the process outcomes as listed above (see item 9).  Data is generated with intention and purpose which shape the type, quality, and content of the information that can be retrieved from it. Therefore, it is important to describe the data collection strategy, the persons who were involved and their qualifications and possible predispositions towards the data collection object, and the particularities and order of procedures, steps, tools and infrastructure used. Furthermore, be aware of the role you have as a researcher in the process evaluation, for example if you have played an important role in the implementation support. The relationship and extent of interaction between the researcher and their participants should be described as it can have an effect on the participants’ responses and also on the researchers’ understanding of the intervention. It might also be that a separate team has conducted the process evaluation data collection and/or analyses to reduce potential bias. If this is the case, this should also be reported. | | |
| 12 | Control group data collection | Describe if any process evaluation data were collected among participants in the control or comparison group(s). |
| **Example:** *We asked both the intervention and the control group participants whether they had participated in any other lifestyle programs during the trial period.*  **Explanation:** In most process evaluations, the primary data collection will be from those who were exposed to the intervention. However, in some cases, data may also be collected from the control or comparison group to provide explanations for potential effects or gain insight into exposure to other interventions besides the one being studies. | | |
| 13 | Process evaluation data analysis | Describe 1) data analysis (i.e. qualitative, quantitative and/or mixed methods); 2) who analysed the data; and 3) if the results of the effectiveness study were known at the time of process data analysis. *If applicable, name and describe the software used to facilitate the analysis. If applicable, describe how data integration techniques were conducted to integrate quantitative and qualitative data.*  *If applicable, describe if the relationship between process evaluation findings and effectiveness outcomes were assessed (e.g. dose-response analysis).* |
| **Example:** *Quantitative data. Descriptive statistics (mean, SD, proportions, range) were used to report participants’, occupational physiotherapists’ and departments’ characteristics as well as self-reported questionnaire data, attendance sheets and logbooks of the occupational physiotherapists. Descriptive statistics and visualizations in Excel were used to explore and report the use of the Activator. All reported suggestions and reasons for adaptations to the intervention and any other answers to open-ended questions were listed and summarized.*  *Qualitative data. The analysis followed a direct content analysis approach whereby transcripts were deductively analyzed with a pre-specified codebook for each pre-specified research question. Any text that could not be categorized within the initial coding scheme was given a new open code (inductively). The open codes were eventually sorted and grouped together into different categories and themes following a thematic analysis (Castleberry and Nolen, 2018). Two researchers (JJ and FN) independently coded all qualitative data in Atlas.ti 7.*  *Triangulation and data synthesis. Both qualitative and quantitative data were collected concurrently throughout the DW study and were compared and contrasted to see whether data from qualitative sources could inform observed findings in quantitative data or whether contradictions between data sources were present, with the aim to validate findings across different data sources.*  *Linking implementation to effectiveness outcomes. Associations between individual participant implementation index scores and changes in sitting time and number of step counts were explored using multi-level linear regression analysis in MLwiN 2.22 with a two-level structure (department, individual). Analyses were adjusted for baseline values. Interaction with time was added to obtain the separate effects at four and eight months of follow-up. For all analyses p < 0.05 was regarded statistically significant(40).*  **Explanation:** When reporting on a process evaluation, it is crucial to provide a detailed account of the methods used for analysing the data. Transparency in the different analysis steps will allow for replication, insight in the steps taken, and the trustworthiness of the data (see also item 14). Especially with regard to process evaluations, it is important to report whether results of the effectiveness study were known at the time of process data analysis. This information can influence the way researchers interpret the process evaluation data and may help to predict or explain intervention outcomes. To ensure that the findings are correctly interpreted, it is advisable to report on the chronological order of the analyses.  We have explicitly included the relationship between process evaluation findings and effectiveness outcomes in the reporting guidelines. As process evaluations are often published separately from their effectiveness paper, this can result in lack of understanding of the link between the implementation process and intervention effect. Some studies have added a dose-response analyses to their process evaluation as a first step to explore this causal pathway. However, quantifying the implementation process can be challenging due to its complexity. Nevertheless, gaining insight into the effect of the working mechanisms (as described in item 5 Mechanisms of the intervention) will help to further improve the intervention and allow for its replication beyond a controlled research setting. | | |
| 14 | Quality assurance of data analysis | Describe techniques that were used to ensure the quality of qualitative and/or quantitative data analysis. *If applicable, for qualitative analyses describe processes such as member checking, audit trail, and triangulation. For quantitative analyses describe processes such as data cleaning, blinding, and how missing data were handled.* |
| **Example:** *Qualitative data (study team notes, observations, interviews, focus groups) were analysed following a framework approach, which included the development and testing of a thematic framework through on-going discussion in the research team. The thematic framework was applied separately by researchers in each country in local languages and supported by Nvivo 11, MAXQDA and AtlasTi (depending on site). Data from each of the four sites were then summarised by theme in English by local research teams, with example data extracts translated into English. These summaries were then compared systematically using framework approach matrices by three researchers (CB, NRC and VJP) based on the research questions and research objectives. Qualitative analysts from across the four research teams discussed data extensively and checked interpretations in multiple online and offline fora throughout the analysis process. Finally, to compare findings from different data sources, we were guided by the mixed methods ‘triangulation protocol’, assessing agreement and dissonance across the datasets and also to identify areas of ‘silence’ i.e. where a given dataset has nothing to contribute, summarised in a ‘convergence matrix’ organised by objective. This final stage allowed us to examine the extent to which the data were confirmed, were ambivalent towards or contradicted the causal assumptions in the Theory of change as well as any potential differences in delivery between countries (41).*  **Explanation:** Researchers are often heavily involved in the research process and interact closely with participants, making it difficult to completely eliminate personal bias. To ensure the quality for both quantitative and qualitative data several techniques are available, such as member checking, audit trail, triangulation, data cleaning, blinding, and reporting how missing data were handled. Reporting on these techniques enhances the credibility of the findings by allowing readers the ability to evaluate how these factors may have affected the results and interpretations.  One effective method for ensuring data quality is data triangulation, which involves using a variety of data sources in a study to look at process outcomes. By using multiple sources, findings can be corroborated, and weaknesses in the data can be compensated for by the strengths of other data. This increases the validity and reliability of the results, ultimately leading to more robust conclusions. | | |
| 15 | Characteristics of process evaluation participants | Describe characteristics of participants in the process evaluation, and, where relevant, the intervention deliverer(s) and setting(s), and describe to what degree the process evaluation participants and setting(s) were representative of the target population of the effectiveness trial. *If assessed, describe characteristics of the control or comparison group participants.* |
| **Example:** *Table 3 provides an overview of the characteristics of the eight participating departments that implemented the DW intervention. The size of the departments ranged from 10 to 120 employees, although the larger departments operated mostly in smaller teams. Employees were working in open-plan offices without personal desks. In all departments, except for department #5, employees had the opportunity to work from home for one or two days per week. Work tasks comprised primarily computer-based tasks, but also involved travelling to customers or having internal meetings. Department #5 was confronted with a reorganization at the time of implementation, which resulted in a decrease in number of employees of almost 50% (48–25). The DW intervention was delivered by three male occupational physiotherapists aged between 27 and 61 years with varying work experience (i.e. ranging from recently graduated up to 18 years of work experience) (40).*  **Explanation:** In line with item 10 on recruitment for the process evaluation, this section should report whether recruitment was successful and which participants, intervention deliverer(s) and setting(s) were included in the study. Some of these characteristics may have already been reported as part of the effectiveness evaluation, but it is still useful to report this data in the process evaluation article. It should be explicitly stated whether all trial participants were included in the process evaluation or only a specific group. This information provides transparency and context for the study and allows readers to better understand the population and setting under investigation. A visualisation or table of the participants might be of added value. | | |
| 16 | Results of process evaluation | Describe the results of the process evaluation as defined in the methods section (as listed in items 9 and 13). |
| **No example provided.**  No examples are provided, as the appropriate reporting depends on the specific study context, study design, and methodological choices, and providing generic examples could be misleading.  **Explanation:** The results section of a process evaluation should include a description of the outcome(s) as outlined in item 9 and the recruitment process as outlined in item 10. The format in which the results are presented may vary depending on the journal’s requirements. However, it is important to provide a clear and concise summary of the data collected, including any significant findings, trends or patterns observed. It is important to ensure that the results are presented in a transparent and objective manner, and that the conclusion drawn are supported by the data. | | |
| 17 | Interpretation of findings | Describe how the findings of the process evaluation can be interpreted.  *If applicable, describe how effectiveness outcomes should be interpreted in light of the process evaluation findings.* |
| **No example provided.**  No examples are provided, as the appropriate reporting depends on the specific study context, study design, and methodological choices, and providing generic examples could be misleading.  **Explanation:** Any scientific discourse should critically reflect on the applied methodology and methods, and their consequences for the data and results presented in the study, also in light of the existing literature. It can be helpful to critically reflect on the (methodological) limitations and strengths of your findings and put them in perspective in regard to other research. This is not an exercise of humbleness about the study or findings, but a fundamental principle of good scientific conduct and a key academic responsibility. Besides a discussion of the process evaluation findings in light of the effectiveness outcomes, it should also discuss any (mathematical) assumptions and dispositions the researchers and participants might have had towards the process evaluation. Ultimately (and explicitly), this reflection contributes to a better understanding of the generalisability of the findings to other populations or settings. | | |
| 18 | Unexpected factors | Describe previous unknown factors that may have influenced the implementation of the intervention and/ or the process evaluation results. |
| **Example:** *Other barriers for the development were the COVID-19 lockdown and change of working structure in the organisation. Therefore, the findings of this study should be treated with caution as the circumstances with COVID-19 and organizational alterations limits the generalizability.*  **Explanation:** Unexpected factors or events can be interpreted in (at least) two ways. First, the intervention leads to outcomes that were not expected, and secondly factors such as facilitators or barriers that influenced intervention delivery or implementation can also play a role. In process evaluations, it is important to report any unexpected events or factors that have influenced the delivery or implementation of the intervention. Unexpected factors or events that influenced delivery or implementation of the intervention are relevant to report because they can have had a (major) positive or negative impact on specific process evaluation objectives or stakeholder participation. These should be considered in future research or implementation activities to be able to manage them properly. The factors may have already been assessed as part of the evaluation of influential factors (listed as process evaluation outcomes under item 9) – i.e. facilitators and barriers for implementation. In that case, these factors do not need to be reported again in the discussion section. Yet, a reflection on the likelihood of (re)occurrence and impact in other settings is most helpful to other researchers or stakeholders, as well as suggestions of any potential mitigating actions that were or can be undertaken to manage the factors well. | | |
| 19 | Implications | Discuss implications of the process evaluation outcomes for future intervention delivery, and implications for policy, practice and/or research. |
| **No example provided.**  No examples are provided, as the appropriate reporting depends on the specific study context, study design, and methodological choices, and providing generic examples could be misleading.  **Explanation:** The findings of your process evaluation not only provide (partial) answers to process evaluation aim(s) and/or objective(s), but they can also confirm or recommend changes to current practice and policy making. Additionally, the findings can inform ongoing or new research, intervention development and adoption (i.e. decision making) and/ or implementation processes. To enable this, it is important to discuss your findings in light of existing practices and policies. By doing so, future research can build on your findings. It is beneficial to address different interest groups explicitly in the discussion section (such as practice, policy and research), providing tailored action-oriented recommendations based on the findings, rather than a description or summary. | | |
