# Supplementary material for: CONsensus-based Process evaluation reporting guideline for public HEalth intervention Studies (CONPHES) conducted alongside an effectiveness trial: an e-Delphi study
Source: BMJ Open. 2025 Dec 19;15(12):e093962. doi: 10.1136/bmjopen-2024-093962 (PMC12716498; doi:10.1136/bmjopen-2024-093962)
Supplement: Supplementary file 3 [file bmjopen-2024-093962.reviewer_comments.pdf]

PEER REVIEW HISTORY

BMJ Open publishes all reviews undertaken for accepted manuscripts. Reviewers are asked to complete a checklist review form and are provided with free text boxes to elaborate on their assessment. These free text comments are reproduced below.

ARTICLE DETAILS

Title (Provisional)

CONsensus-based Process evaluation reporting guideline for Public HEalth intervention Studies (CONPHES) conducted alongside an effectiveness trial: An e-Delphi study

Authors

van Nassau, Femke; Cillekens, Bart; Jelsma, Judith; Vis, Christiaan; Mokkink, L.B.; Treweek, Shaun; van der Ploeg, Hidde P; panel members, e-Delphi

---

VERSION 1 - REVIEW

**Reviewer**	**1**
**Name**	**Wabnitz, Katharina**
**Affiliation**	**University of Augsburg Faculty of Medicine**
**Date**	**04-Apr-2025**
**COI**	**None**

Peer review: CONsensus-based Process evaluation reporting guideline for Public HEalth intervention Studies (CONPHES) conducted alongside an effectiveness trial

The aim of the research reported in this manuscript was to develop a guideline for reporting process evaluations of public health interventions, i.e. a guideline for reporting concomitant assessments of mechanisms behind observed effects. The guideline was created based on a virtual consensus development process (e-Delphi study).

I recommend the following revisions (comments 6, 8 and 10 are major):

1. p4, line 53: did 53% complete round 1 of the study or does this number refer to any round?
2. p5, line 50: how was representation achieved? Of whom was the panel representative given the purposive sampling technique? (potentially misleading

wording, recommend deleting representative or elaborate more. This comment also applies to p14, line 25)

3. p6, line 6: following from my first comment, I'd recommend mentioning the share of how many participants completed all rounds before the share of participants per round and mentioning the share of participants per each round for clarity on attrition. I also saw that there's a list of all authors who completed all three rounds in the attachments, however, this is not specified as an Appendix or referred to in the main text which I'd recommend doing
4. p6, line 31: I'd recommend specifying what type of process you refer to – it's clearly defined in the abstract and I'd introduce the paper with this specific definition, too: process evaluations of public health interventions conducted alongside an effectiveness trial
5. p7, line 31: generally, I asked myself why the specification "alongside an effectiveness trial" is needed – I'm assuming the guideline would also be applicable to retrospective evaluations? If not, it might be useful to specify and justify in the text
6. p9, paragraph on scoping review: this section lacks details on how the scoping review was performed, impeding repetition of this part of the research, e.g. inclusion and exclusion criteria for retrieved studies as well as how articles were screened and inclusion decisions reached. Referring to the PRISMA-ScR checklist might be helpful here
7. p9, line 38: in the protocol published on the EQUATOR website, it was stated that a 9-point Likert scale would be used. Here, a 5-point Likert scale is reported. I recommend making this (and others that I might have missed) deviation from the protocol transparent in the manuscript and justify them. I recommend not reporting these changes and their justifications in the Strengths and Limitations section but in the Methods section either as a stand-alone paragraph or along the existing sections as you see fit.
8. p9, e-Delphi procedure: I'd recommend expanding on how free text answers were analysed and changes to items agreed upon within the steering group
9. Results section:

This section lacks details on the results of the scoping review. As the authors claim to fill a gap in the literature, it would be interesting to know more about the 14 guidelines that were found and used as a basis for the e-Delphi process – how do these guidelines not fill the postulated gap? I'd also recommend reflecting on the limitations of the scoping review in the Strengths and Limitations section, e.g. searching one scientific database only

10. p12, line 25: it is unclear why a different "basic population" is being referred to, i.e. 64 instead of 73 (which I understood to be the overall number of panel members).

11. Discussion section:

The discussion should be extended to include reflections on how this guideline differs from existing ones (following on from my comment on the results of the scoping review). It would be interesting to understand why the specification of process evaluations conducted in the context of effectiveness trials was deemed important by panel members. p14, line 11: It is stated that process evaluations can be carried out using diverse methods – as there are reporting guidelines for quantitative studies, qualitative studies and mixed-methods studies available as per the repository of the EQUATOR network, what precisely is the added value of this reporting guideline?

12. Table 2: The specification of what type of process evaluation this study focussed on, namely those conducted alongside an effectiveness trial of public health interventions, is missing from Item 1.

Some copy-editing is needed throughout the manuscript and all appendices, e.g. regarding singular vs plural words (example: p44, line 10: these reporting guidelines should be This reporting guideline)

**Reviewer**	**2**
**Name**	**Prince, Stephanie A.**
**Affiliation**	**Public Health Agency of Canada**
**Date**	**15-Aug-2025**
**COI**	**None**

General comments:

Thank you for the opportunity to review this paper describing a modified Delphi study to develop consensus on a reporting guideline for process evaluations of public health intervention studies. Given the increased prevalence of these types of studies in the literature and the variation in reporting quality, it is important that a guideline be developed to help editors, reviewers and study authors improve reporting quality. The authors should be commended on taking on this task, and for writing a very clear and comprehensive paper. I have no major concerns with the paper, but have some suggestions for improved clarity.

I am providing some specific points both major and minor below:

Major points:

1. Page 12, line 30: Can the authors expand on how and what was included in the final decision made re: pending item description after round 3?

2. Is the intention that study authors would consult other reporting guidelines for best practices on other evaluation information to report e.g., participant characteristics? Item 3

has “target population”, but not reporting of the participants or flow. I read that it was suggested by Delphi participants to consider removing items that already appeared in general reporting guidelines or are part of general reporting practices, but it may be good to include a note about this somewhere on the checklist. I wanted to note, I see this elaborated on in the E&E document, but it might be good to flag this upfront in the guide itself.

3. Page 14, line 25: how was this panel “representative”? Consider removing this term especially as there was likely underrepresentation from LMICs.

4. Page 14, line 58: explain why the 67% was chosen vs the previous 80%?

5. Did the Delphi participants also review the final manuscript for inclusion as authors or will they appear in an appendix list only?

Minor points:

1. Abstract: I don’t think that (E&E) is necessary as it’s a single occurrence.

2. Page 6, line 33: Is there a citation for the PubMed findings provided?

3. Page 6, line 51: I think there is an extra word here (to).

4. In the Introduction, consider defining an effectiveness trial.

5. Should this study be referred to as a modified e-Delphi study as the study began with a pre-defined list for feedback rather than open ended questions to generate items?

6. Why was 67% used as the cut-off for consensus?

7. Page 10, line 49: The sentence structure needs to be changes to improve readability. E.g., …85% were occupied as a health research, 23% as a methodologist, 10% as a journal editor…

8. For the Appendix 3 figure, consider adding round descriptions in terms of what was conducted within each round.

9. Page 12, line 5: items should be pluralized

10. Are the authors aware of any MESH terms that would be useful to include as keywords to capture process evaluations? It may be something to consider in the description for item 1.

11. Could examples be provided for item 8?

12. Item 3, I think there’s a minor typo in the description, should the e.g. be present? “…with reference to previous research, frameworks…”

13. Line 40 in the Appendix, should this be in a clear vs. on a clear?

14. In the E&E document, item 11: should the example be written as it would appear in a publication? E.g., qualitative data was collected…were conducted…

15. Why is no example provided for items16 and 17 in the E&E document?

16. Page 16, line 45: thanks should not be pluralized.

17. Table 1 footnotes identified n=8 for Canada, but only 4 authors have Canadian affiliations. Suggest double-checking these numbers.

VERSION 1 - AUTHOR RESPONSE

Reviewer 1

The aim of the research reported in this manuscript was to develop a guideline for reporting process evaluations of public health interventions, i.e. a guideline for reporting concomitant assessments of mechanisms behind observed effects. The guideline was created based on a virtual consensus development process (e-Delphi study).

Response:

We sincerely thank the reviewer for carefully reviewing our manuscript and providing valuable feedback. We greatly appreciate the constructive comments, which have helped us to improve the clarity and quality of our work. Below, we address each point raised by the reviewer in a point-by-point manner.

1. **Comment**

   p4, line 53: did 53% complete round 1 of the study or does this number refer to any round?

1.1. Response:

We thank the reviewer for this question. A total of 73 participants took part in at least one Delphi round. Most participants joined in round 1 (n = 72), while one participant indicated a preference to join later and therefore started from round 2 onwards. We believe the current text accurately reflects this information. However, to clarify participation across rounds, we have ensured that the Methods and Results sections and the flowchart in the Appendix 3 now provide a more detailed explanation of participant numbers per round.

2. **Comment**

   p5, line 50: how was representation achieved? Of whom was the panel representative given the purposive sampling technique? (potentially misleading wording, recommend deleting representative or elaborate more. This comment also applies to p14, line 25)

2.1. Response:

We thank the reviewer for highlighting this point. To avoid potential misunderstanding, we have removed the word “representative”. We now describe the panel as a purposively sampled group of

international experts in public health, process evaluation, and clinical trials. Additionally, we have addressed this as a limitation in the Discussion section that participation from experts based in low- and middle-income countries (LMICs) was relatively low.

In the results section, we now added: (Page 11, line 3-7):

"Of the 73 participants who took part in at least one Delphi round, the vast majority were based in Europe (65%) and North America (23%), with a smaller number from Oceania (8%). Participation from Africa and Asia was limited (4%). This geographical distribution reflects the composition of the expert panel and is noted as a limitation in the discussion."

In the discussion section we now added this paragraph (Page 15, line 22 to page 16, line 2):

"A limitation of our study is the geographical distribution of the expert panel. Of the 73 participants who took part in at least one e-Delphi round, the vast majority were based in Europe and North America, with a smaller number from Oceania. Participation from Africa and Asia was limited, representing less than 5% of the total panel. While the panel included diverse professional backgrounds, the underrepresentation of experts from low- and middle-income countries may affect the generalizability of the guideline to these settings."

3. Comment:

p6, line 6: following from my first comment, I'd recommend mentioning the share of how many participants completed all rounds before the share of participants per round and mentioning the share of participants per each round for clarity on attrition. I also saw that there's a list of all authors who completed all three rounds in the attachments, however, this is not specified as an Appendix or referred to in the main text which I'd recommend doing

3.1. Response:

We thank the reviewer for this helpful suggestion. We have revised the text to clearly indicate that the panel members were highly engaged in the consensus process.

The text now reads (Page 10, line 16-18):

"Of the 137 experts invited, 72 participated in round 1, 64 in round 2 (including 1 participant who did not take part in round 1), and 55 completed round 3 (67%) – who are listed as e-Delphi panel members."

4. **Comment**

p6, line 31: I'd recommend specifying what type of process you refer to – it's clearly defined in the abstract and I'd introduce the paper with this specific definition, too: process evaluations of public health interventions conducted alongside an effectiveness trial

4.1. **Response:**

We thank the reviewer for this suggestion. The first sentence (line 31) of the Introduction has been left unchanged, as it was carefully formulated to align with the search strategy for process evaluations in Pubmed. However, in the first paragraph, we have added a clarifying sentence to explicitly define the type of process evaluation addressed in this study. See page 6, line 3-4.

5. **Comment**

p7, line 31: generally, I asked myself why the specification "alongside an effectiveness trial" is needed – I'm assuming the guideline would also be applicable to retrospective evaluations? If not, it might be useful to specify and justify in the text

5.1. **Response:**

We thank the reviewer for raising this important point. We deliberately focused on process evaluations conducted alongside effectiveness trials to define a clear and specific scope for the guideline, as this niche reflects the majority of the studies considered and the expertise of our e-Delphi panel. However, we acknowledge that the CONPHES guideline may also be relevant to other types of process evaluations, including retrospective evaluations. We have added a note in the Discussion section to clarify this and to indicate that while the guideline was developed with prospective, alongside-trial evaluations in mind, its principles can be applied more broadly. See page 14, line 8-9:

"Furthermore, the guideline can also be relevant to other types of process evaluations, including retrospective evaluations."

6. **Comment**

p9, paragraph on scoping review: this section lacks details on how the scoping review was performed, impeding repetition of this part of the research, e.g. inclusion and exclusion criteria for retrieved studies as well as how articles were screened and inclusion decisions reached. Referring to the PRISMA-ScR checklist might be helpful here

6.1. **Response:**

We thank the reviewer for this comment. We have revised the text to clarify our approach. Rather than a full scoping review, we conducted a literature search with the aim of identifying existing checklists used to assess or review process evaluation studies, potential reporting guidelines for

process evaluations, and systematic reviews of process evaluation studies. From this search, we identified 14 relevant guidelines, from which we extracted potential domains to inform the development of our own CONPHES guideline. We have clarified this methodology in the manuscript and removed the term "scoping review" to more accurately reflect the nature of our approach. See page 8, line 2-4:

"a literature search to identify existing checklists used to assess or review process evaluation studies, potential reporting guidelines for process evaluations, and systematic reviews of process evaluation studies."

7. **Comment**

p9, line 38: in the protocol published on the EQUATOR website, it was stated that a 9-point Likert scale would be used. Here, a 5-point Likert scale is reported. I recommend making this (and others that I might have missed) deviation from the protocol transparent in the manuscript and justify them. I recommend not reporting these changes and their justifications in the Strengths and Limitations section but in the Methods section either as a standalone paragraph or along the existing sections as you see fit.

7.1. Response:

We mentioned changes to the published protocol in our discussion section, but have now moved this as suggested by the reviewer to the methods section. See page 7, line 15-18:

"We did make some changes to the published protocol: we applied a 67% (vs 80%) threshold for consensus in line with other studies (20), and used a 5-point Likert scale instead of a 9-point scale. We were also able to recruit more panel members than anticipated (73 vs. the 30 we had planned)."

8. **Comment**

p9, e-Delphi procedure: I'd recommend expanding on how free text answers were analysed and changes to items agreed upon within the steering group

8.1. Response:

We thank the reviewer for this helpful suggestion. We have added clarification in the manuscript: Free-text responses were initially analyzed independently by FN and BC, who extracted and grouped the main points. Remaining discussion points were then presented to the steering group for deliberation and agreement on any changes to the items. In the subsequent e-Delphi round, participants were informed of the changes and provided with explanations regarding how their feedback had been incorporated.

***We have added this to the main manuscript, this now reads*: (page 9, line 21-25)**

"Free-text responses were initially analyzed independently by FN and BC, who extracted and grouped the main points. Remaining discussion points were then presented to the steering group for deliberation and agreement on any changes to the items. In the subsequent Delphi round, participants were informed of the changes and provided with explanations regarding how their feedback had been incorporated."

9. Comment

Results section: This section lacks details on the results of the scoping review. As the authors claim to fill a gap in the literature, it would be interesting to know more about the 14 guidelines that were found and used as a basis for the e-Delphi process – how do these guidelines not fill the postulated gap? I'd also recommend reflecting on the limitations of the scoping review in the Strengths and Limitations section, e.g. searching one scientific database only

9.1. Response:

We thank the reviewer for this suggestion. We have clarified in the manuscript that a literature search (rather than a full scoping review – see comment 6) was conducted to identify existing guidelines and checklists relevant to process evaluations. From this search, we identified 14 guidelines, which we have now either cited in the manuscript. These existing guidelines did not comprehensively cover the specific reporting needs for process evaluations conducted alongside effectiveness trials, which justified the development of the CONPHES guideline.

See page 9, line 3:

"In total, 14 guidelines were found (9, 11-15, 21-28)."

10. Comment

p12, line 25: it is unclear why a different "basic population" is being referred to, i.e. 64 instead of 73 (which I understood to be the overall number of panel members).

10.1. Response

The number 64 refers specifically to the participants who took part in round 2 of the Delphi study. The overall number of panel members who participated in at least one round was 73. We have clarified this in the manuscript to avoid confusion regarding the "basic population" for each round.

We changes the text in the manuscript to make it more clear: page 10, line 16-18:

"Of the 137 experts invited, 72 participated in round 1, 64 in round 2 (including 1 participant who did not take part in round 1), and 55 completed round 3 (67%) – who are listed as e-Delphi panel members."

11. **Comment**

Discussion section: The discussion should be extended to include reflections on how this guideline differs from existing ones (following on from my comment on the results of the scoping review).

11.1. **Response:**

We thank the reviewer for this suggestion. We already addressed this in our discussion as follows (page 14, line 14-18):

"And no available reporting guidelines were available. We also did not aim to replace existing guidelines and recognise that some parts of the reporting guideline can be complemented with other existing guidelines, such as TIDieR Checklist with regard to intervention descriptions (11) or the Standards for Reporting Implementation Studies (StaRI) (15). We, therefore, have referred to such guidelines in the E&E document."

12. **Comment**

It would be interesting to understand why the specification of process evaluations conducted in the context of effectiveness trials was deemed important by panel members.

12.1. **Response:**

We thank the reviewer for raising this question. The rationale for developing this guideline stemmed from our experience conducting many process evaluations alongside effectiveness trials, where we found no suitable reporting guideline. A literature search confirmed this gap. We therefore initiated the e-Delphi study, which was shaped by this scope. The high participation rate and the feedback we received from the panel further underscored both the relevance of the gap and the added value of developing these guidelines alongside existing ones.

13. **Comment**

p14, line 11: It is stated that process evaluations can be carried out using diverse methods – as there are reporting guidelines for quantitative studies, qualitative studies and mixed-methods studies available as per the repository of the EQUATOR network, what precisely is the added value of this reporting guideline?

13.1. **Response:**

While existing guidelines cover specific study designs (quantitative, qualitative, or mixed-methods), CONPHES provides a consensus-based framework specifically for process evaluations conducted alongside effectiveness trials of public health interventions. Its added value lies in addressing the unique combination of reporting on implementation, intervention mechanisms, and contextual factors that are often not fully captured by other design-specific guidelines. CONPHES is intended

to complement, rather than replace, existing reporting guidance, ensuring that process evaluations are reported consistently, transparently, and in a manner directly relevant to parallel effectiveness trials.

14. **Comment:**

Table 2: The specification of what type of process evaluation this study focussed on, namely those conducted alongside an effectiveness trial of public health interventions, is missing from Item 1.

14.1. **Response:**

We thank the reviewer for this observation. As the items in Table 2 reflect the outcome of a consensus-based Delphi process, we are unable to modify them. However, we have clarified this point in the Explanation & Elaboration (E&E) document, where the scope and context of the process evaluations addressed by CONPHES are explicitly described.

15. **Comment**

Some copy-editing is needed throughout the manuscript and all appendices, e.g. regarding singular vs plural words (example: p44, line 10: these reporting guidelines should be This reporting guideline)

15.1. **Response:**

We have double checked our manuscript on writing.

Reviewer 2:

1. **General comments:**

   Thank you for the opportunity to review this paper describing a modified Delphi study to develop consensus on a reporting guideline for process evaluations of public health intervention studies. Given the increased prevalence of these types of studies in the literature and the variation in reporting quality, it is important that a guideline be developed to help editors, reviewers and study authors improve reporting quality. The authors should be commended on taking on this task, and for writing a very clear and comprehensive paper. I have no major concerns with the paper, but have some suggestions for improved clarity.

1. **Response:**

   We sincerely thank the reviewer for their careful reading of our manuscript and their positive and constructive feedback. We very much appreciate the acknowledgement of the importance of developing this reporting guideline and of the clarity and

comprehensiveness of our paper. We are grateful for the helpful suggestions provided and have addressed each of them in detail below.

2. **Comment**

   Page 12, line 30: Can the authors expand on how and what was included in the final decision made re: pending item description after round 3?

2.1. Response:

We thank the reviewer for this helpful comment and understand that our original description was not sufficiently clear. Consensus was reached on all 19 items; however, for one description (item 18: unexpected factors), consensus was not reached (60% instead of >67%). The steering group therefore discussed the remaining comments and suggestions, and the description was revised accordingly.

The original description was:
"Describe unexpected factors that may have influenced the implementation of the intervention during the effectiveness study."
Based on the panel's feedback, this was changed into:
"Describe previously unforeseen factors that may have influenced the implementation of the intervention and/or the process evaluation results."

The revised text in the manuscript now reads: (see page 12, line 20-23)
"Consensus was reached on all item definitions and descriptions, except for the description of one item (item 18: unexpected factors; see appendix 6), where 60% of panel members agreed. The steering group discussed the remaining feedback and suggestions for this item after round 3 and revised the description accordingly."

3. **Comment:**

   Is the intention that study authors would consult other reporting guidelines for best practices on other evaluation information to report e.g., participant characteristics? Item 3 has "target population", but not reporting of the participants or flow. I read that it was suggested by Delphi participants to consider removing items that already appeared in general reporting guidelines or are part of general reporting practices, but it may be good to include a note about this somewhere on the checklist. I wanted to note, I see this elaborated on in the E&E document, but it might be good to flag this upfront in the guide itself.

3.1. Response:

As noted, this issue is already addressed in the E&E document, where we explain that some aspects (e.g., participant characteristics, participant flow) are covered by general reporting guidelines and were therefore not duplicated in CONPHES. We believe that referring authors to the E&E document is sufficient and avoids overlap or redundancy within the checklist itself. Ultimately, it is also up to journals to decide how such complementary use of multiple reporting guidelines is to be applied in practice.

4. **Comment:**

   Page 14, line 25: how was this panel "representative"? Consider removing this term especially as there was likely underrepresentation from LMICs.

4.1. **Response:**

   We agree with the concern and have removed the term "representative" from the manuscript. As also noted in our response to Reviewer 1 (comments 2), we have additionally acknowledged the limited participation from LMICs as a study limitation in the Discussion section.

In the results section, we now added: (Page 11, line 3-7):
"Of the 73 participants who took part in at least one Delphi round, the vast majority were based in Europe (65%) and North America (23%), with a smaller number from Oceania (8%). Participation from Africa and Asia was limited (4%). This geographical distribution reflects the composition of the expert panel and is noted as a limitation in the discussion."

In the discussion section we now added this paragraph (Page 15, line 22 to page 16, line 2):
"A limitation of our study is the geographical distribution of the expert panel. Of the 73 participants who took part in at least one e-Delphi round, the vast majority were based in Europe and North America, with a smaller number from Oceania. Participation from Africa and Asia was limited, representing less than 5% of the total panel. While the panel included diverse professional backgrounds, the underrepresentation of experts from low- and middle-income countries may affect the generalizability of the guideline to these settings."

5. **Comment:**

   Page 14, line 58: explain why the 67% was chosen vs the previous 80%?

5.1. **Response:**

   We thank the reviewer for this question. In the original protocol and funding plan, an 80% threshold was initially proposed. However, after consulting our expert in Delphi studies for this project (co-author Mokkink), we decided to use ≥67% (two-thirds) as the consensus

threshold. We chose this lower threshold because we view consensus as a means to assess agreement and guide item development, rather than a goal in itself. A two-thirds threshold allows participants the space to express critical perspectives, encouraging discussion and refinement of items rather than forcing full agreement. While the choice of threshold is ultimately arbitrary, this approach helps ensure that the guideline is robust and critically evaluated by the expert panel (1). No changes were made to the manuscript.

(1) Diamond IR, Grant RC, Feldman BM, Pencharz PB, Ling SC, Moore AM, Wales PW. Defining consensus: a systematic review recommends methodologic criteria for reporting of Delphi studies. J Clin Epidemiol. 2014 Apr;67(4):401-9. doi: 10.1016/j.jclinepi.2013.12.002. PMID: 24581294.

6. **Comment**

Did the Delphi participants also review the final manuscript for inclusion as authors or will they appear in an appendix list only?

6.1. **Response:**

The Delphi participants are listed in the Appendix only. Participants were not included as co-authors but are acknowledged as group author in the Appendix for their contributions to the Delphi process, and therefore were informed about the final manuscript, but not asked to provide feedback. As their feedback was already incorporated in the e-Delphi rounds.

7. **Comment:**

Abstract: I don't think that (E&E) is necessary as it's a single occurrence.

7.1. **Response:**

We have deleted the abbreviation.

8. **Comment:**

Page 6, line 33: Is there a citation for the PubMed findings provided?

8.1. **Response**

No, but we have now included the references in the methods section.

See page 9, line 3:

"In total, 14 guidelines were found (9, 11-15, 21-28)."

9. **Comment:**

Page 6, line 51: I think there is an extra word here (to).

9.1. Response:

Thanks, we deleted (to)

10. Comment

In the Introduction, consider defining an effectiveness trial.

10.1. Response:

We have clarified this as following: Page 6, line 11:

"looking at the intervention effect on the target population."

11. Comment:

Should this study be referred to as a modified e-Delphi study as the study began with a pre-defined list for feedback rather than open ended questions to generate items?

11.1. Response:

We thank the editor for raising this point. We are happy to use the term "modified e-Delphi study" if it is deemed more appropriate, although we note that there are no strict rules requiring a Delphi study to begin with open-ended questions. Our main aim was to ensure clarity that a Delphi methodology was applied, and we are comfortable adapting the terminology to the journal's preference.

12. Comment:

Why was 67% used as the cut-off for consensus?

12.1. Response:

We addressed this comment at comment 5.

13. Comment:

Page 10, line 49: The sentence structure needs to be changes to improve readability. E.g., …85% were occupied as a health research, 23% as a methodologist, 10% as a journal editor…

13.1. Response:

We thank the reviewer for this helpful suggestion. We have revised the sentence for clarity and readability. The text now reads: (page 10, line 22 to page 11, line 3)

"With regard to professional background, 85% of participants were health researchers, 23% methodologists, 10% journal editors, and 4% guideline developers."

14. Comment:

For the Appendix 3 figure, consider adding round descriptions in terms of what was conducted within each round.

14.1. Response:

We thank the reviewer for this suggestion. While we understand the interest in providing detailed descriptions of each round, including this level of detail in the manuscript or appendix would be excessive. For reference, the feedback report from round 1 contained 106 pages, round 2 approximately 50 pages, and round 3 fewer. We are happy to provide this detailed information to interested readers upon request.

15. Comment

Page 12, line 5: items should be pluralized

15.1. Response:

We adjusted this.

16. Comment

Are the authors aware of any MESH terms that would be useful to include as keywords to capture process evaluations? It may be something to consider in the description for item 1.

16.1. Response:

We thank the reviewer for this suggestion. We are aware of relevant MeSH terms; however, as item 1 and its description were finalized through the consensus process, we are unable to modify these items or descriptions.

17. Comments

Could examples be provided for item 8?

17.1. Response:

Examples are provided in the E&E.

18. Comment:

Item 3, I think there's a minor typo in the description, should the e.g. be present? "…with reference to previous research, frameworks…"

18.1. Response:

We thank the reviewer for pointing this out. The "e.g." is intentional in the description, as it indicates that previous research, frameworks, and co-creation are examples of sources

that may have informed the intervention development process. No change has been made to the item description.

19. **Comment**

Line 40 in the Appendix, should this be in a clear vs. on a clear?

19.1. Response:

We have adjusted this.

20. **Comment**

In the E&E document, item 11: should the example be written as it would appear in a publication? E.g., qualitative data was collected…were conducted…

20.1. Response:

Done.

21. **Comment**

Why is no example provided for items16 and 17 in the E&E document?

21.1. Response

No examples are provided for items 16 and 17 because their content depends heavily on the specific study context, choices made, and study type. Providing generic examples could therefore be misleading. We have clarified this rationale in the E&E document.

22. **Comment**

Page 16, line 45: thanks should not be pluralized.

22.1. Response:

We have adjusted this.

23. **Comments**

Table 1 footnotes identified n=8 for Canada, but only 4 authors have Canadian affiliations. Suggest double-checking these numbers.

23.1. Response:

We thank the reviewer for pointing this out. The discrepancy arises because Table 1 reflects all 73 participants who took part in at least one Delphi round, whereas the acknowledgements list includes only participants who completed all rounds. Additionally, some participants have multiple affiliations or have changed affiliations since completing round 1.

VERSION 2 - REVIEW

**Reviewer**	**1**
**Name**	**Wabnitz, Katharina**
**Affiliation**	**University of Augsburg Faculty of Medicine**
**Date**	**27-Oct-2025**
**COI**	

Thank you for submitting a revised version of your manuscript. I deem the peer review comments sufficiently addressed.

**Reviewer**	**2**
**Name**	**Prince, Stephanie A.**
**Affiliation**	**Public Health Agency of Canada**
**Date**	**29-Oct-2025**
**COI**	

Thank you to the authors for considering the reviewer feedback. All concerns have been adequately addressed.
